# Efficacy and safety of perospirone as adjunctive therapy in major depressive disorder patients with inadequate response to antidepressants: a randomized clinical trial

**DOI:** 10.1016/j.eclinm.2025.103626

**Published:** 2025-11-07

**Authors:** Jin Liu, Sirui Gao, Bangshan Liu, Yumeng Ju, Mei Liao, Li Zhang, Zexuan Li, Qianqian Zhang, Xiaotian Zhao, Hao Tang, Ruhong Jiang, Jian Zhou, Danfeng Yan, Zhiren Wang, Yixiong Zheng, Duanfang Cai, Ning Wei, Yunshu Zhang, Han Yu, Lijun Ding, Binhong Wang, Guangya Liu, Keqing Li, Shaohua Hu, Fude Yang, Li Kuang, Zhijian Yao, Yan Zhang, Lingjiang Li

**Affiliations:** a Department of Psychiatry, National Clinical Research Center for Mental Disorders, and National Center for Mental Disorders, The Second Xiangya Hospital of Central South University, Changsha, Hunan, China; b Department of Psychiatry, The Affiliated Brain Hospital of Nanjing Medical University, No. 264 Guangzhou Road, Nanjing, 210029, China; c Department of Psychiatry, The First Affiliated Hospital of Chongqing Medical University, 1 Youyi Road, Yuzhong District, Chongqing, 400016, China; d Department of Psychiatry, Brains Hospital of Hunan Province, Changsha, PR China; e Department of Psychiatry, Shanxi Mental Health Center, Taiyuan Psychiatric Hospital, School of Mental Health, Shanxi Medical University, Mental Health Hospital of Shanxi Medical University, No. 55 Nanshifang St., Yingze District, Taiyuan City, 030045, Shanxi, China; f Peking University HuiLongGuan Clinical Medical School, Beijing Huilongguan Hospital, Beijing, China; g School of Clinical Medicine, Fujian Medical University, Fuzhou, Fujian, China; h Department of Psychiatry, Zigong Mental Health Center, The Zigong Affiliated Hospital of Southwest Medical University, Zigong, Sichuan Province, China; i Department of Psychiatry, The First Affiliated Hospital, Zhejiang University School of Medicine, Hangzhou 310003, China; j Hebei Provincial Mental Health Center, No. 572, Dongfeng East Rd., Baoding City, Hebei Province, China

**Keywords:** Major depressive disorder, Adjunctive therapy, Second-generation antipsychotic, Perospirone, Early inadequate response

## Abstract

**Background:**

Adjunctive therapy with a second-generation antipsychotic (SGA) represents a treatment option for major depressive disorder (MDD) patients with inadequate response to antidepressants. Evidence suggests that early initiation of adjunctive SGA treatment may yield greater benefits. This study aimed to investigate the efficacy, safety, and tolerability of adjunctive therapy with perospirone initiated after 4 weeks of antidepressant treatment in MDD patients with inadequate response.

**Methods:**

This phase IV, multi-center, randomized, double-blind, placebo-controlled trial assessed the efficacy, safety and tolerability of perospirone as an adjunct to serotonin norepinephrine reuptake inhibitors (SNRIs) or selective serotonin reuptake inhibitors (SSRIs) in patients with MDD who showed inadequate response to SSRIs/SNRIs. Patients with moderate-to-severe depression (defined as a baseline Montgomery-Åsberg Depression Rating Scale [MADRS] score of ≥20) who exhibited a reduction of <50% in the total score of MADRS after receiving at least one antidepressant at an adequate dose for at least four weeks during the current episode were included in the study. The primary intervention timepoint was set at week 4, with treatment initiation permitted up to week 8. Eligible patients were randomized 1:1 to the perospirone group (perospirone + SSRIs/SNRIs) or the placebo group (placebo + SSRIs/SNRIs) and received treatment for 8 weeks. Perospirone was administered once or twice daily at an initial dose of 4–8 mg/day and a maintenance dose of 12–48 mg/day, based on symptom severity and patient tolerance. The study, conducted across 10 Chinese hospitals between December 16, 2021 and June 19, 2024, evaluated the primary efficacy endpoints of response (≥50% reduction in the MADRS score) and remission (MADRS score ≤10) rates at the end of the 8-week treatment. The study was registered at www.chictr.org.cn (ChiCTR2200063354).

**Findings:**

A total of 210 participants were randomized into the two groups (108 were assigned to the perospirone group and 102 to the placebo group). Compared to the placebo group, more patients in the perospirone group achieved remission at week 8 (55.2% vs. 38.9%, odds ratio [OR] = 1.944, 95% confidence interval [CI]: 1.060–3.564, p = 0.032), and the proportions of participants with clinical response were comparable between the two groups (67.8% vs. 60.0%, OR = 1.406, 95% CI: 0.759–2.605, p = 0.28). Most common adverse events (AEs) were mild arousal-related symptoms, autonomic dysfunction, and extrapyramidal symptoms. Treatment discontinuation rates due to AEs were low and comparable in both groups, with no significant safety concerns.

**Interpretation:**

Early adjunctive treatment with perospirone for 8 weeks may confer potential benefits, particularly in MDD patients who exhibit inadequate response to SSRIs/SNRIs and present with more severe symptoms requiring rapid improvement. While the treatment demonstrated acceptable tolerability and safety profiles, the observed efficacy should be interpreted with caution and regarded as hypothesis-generating due to the lack of multiplicity adjustment. Further confirmatory randomized controlled trials are warranted to validate these findings.

**Funding:**

The study was supported by the STI2030-Major Projects (grant 2021ZD0202000), 10.13039/501100001809National Natural Science Foundation of China (grant 82201693 and 82471555), Hunan Provincial Natural Science Outstanding Youth Foundation (grant 2025JJ20093), Science and Technology Innovation Program of Hunan Province (grant 2024RC3057), 10.13039/501100012151Sanming Project of Medicine in Shenzhen (grant SZSM202311025), and Reboscience (Zhuhai) Pharmaceutical Research Co., Ltd., and sponsored by Livzon Pharmaceutical Group Inc.

**Research in context**

**Evidence before this study**

“Perospirone”, “Depression”, “Inadequate response”, “Treatment-resistant”, “Antipsychotic”, and “Augmentation” were used as search terms in PubMed, Embase, and Cochrane Library up to January 1, 2025. Preclinical evidence from two animal studies revealed that perospirone combined with antidepressants elevated dopamine levels in the rat medial prefrontal cortex, suggesting potential antidepressive mechanisms. However, clinical evidence remains limited, only one open-label pilot study has preliminarily investigated the efficacy of perospirone in treatment-resistant depression, which was defined in that study as incomplete or absent response to various antidepressant monotherapies or combination therapies for eight weeks or longer. No prior randomized controlled trials have established its effectiveness in patients with major depressive disorder (MDD) who exhibit inadequate response to at least one antidepressant after a minimum of four weeks of adequate-dose treatment during the current episode.

**Added value of this study**

This multi-center, randomized, double-blind, placebo-controlled trial provides the first preliminary evidence for early perospirone adjunctive therapy in MDD patients with inadequate response to antidepressants. The findings suggest that 8 weeks of adjunctive therapy with perospirone may confer potential benefits in improving treatment outcomes, with favorable safety and tolerability profiles.

**Implications of all the available evidence**

The current study strengthens existing evidence by suggesting that early 8-week adjunctive therapy with perospirone may represent a potentially beneficial and well-tolerated therapeutic strategy for MDD patients with inadequate response to antidepressants after at least 4 weeks of treatment. These findings suggest that the adjunctive therapy with perospirone represents a feasible option for MDD patients with inadequate response to antidepressants, thereby providing a potentially effective strategy to improve treatment outcomes in this patient population.

## Introduction

To date, antidepressants such as selective serotonin reuptake inhibitors (SSRIs) and serotonin norepinephrine reuptake inhibitors (SNRIs) are the preferred first-line treatment for patients with major depressive disorder (MDD). However, the Sequenced Treatment Alternatives to Relieve Depression (STAR∗D) study found that only 28% of the patients achieved remission after an initial treatment with SSRIs.^1^ According to previous meta-analyses, among patients who received antidepressant monotherapy, approximately 40–60% were responsive and only 20–40% achieved clinical remission during their treatment.^2^^,^^3^ For patients with inadequate response to SSRIs/SNRIs, meta-analyses of randomized, double-blind, placebo-controlled trials have demonstrated that the adjunctive use of second-generation antipsychotics (SGAs) along with SSRIs/SNRIs can significantly improve patient response, with pooled estimates showing consistent efficacy across studies.^1^, ^2^, ^3^ The adjunctive use of SGA is also recommended in current guidelines.^4^^,^^5^

Although a standard and adequate duration of antidepressant treatment is generally considered to be 8 weeks,^5^^,^^6^ this does not mean that patients should maintain the same regimen throughout the entire period in the absence of early response. As early response is predictive of better treatment and functional outcomes, treatment should be optimized early to improve early response, rather than delaying adjustments until the regimen has failed 8 weeks later.^7^, ^8^, ^9^ Current authoritative international guidelines, including those from the Canadian Network for Mood and Anxiety Treatments (CANMAT) and the Royal Australian and New Zealand College of Psychiatrists (RANZCP), recommend reassessment of the effect of antidepressants after four weeks of treatment; it is also suggested that the SGA adjunctive therapy may be considered if the patient response to the antidepressant is suboptimal at a established therapeutic dose.^4^^,^^5^ This timeframe is also supported by evidence that the absence of early improvement within 2–4 weeks of antidepressant initiation may warrant modification of the management strategy (e.g., adjunctive treatment with an SGA) to prevent treatment resistance.^10^, ^11^, ^12^ However, although guidelines and studies have recommended a timeframe of 4 weeks before the initiation of SGA adjunctive therapy, the majority of current trials adopted an 8-week (or at least 6-week) timeframe before initiating the SGA adjunctive therapy.^13^, ^14^, ^15^, ^16^ Thus, it remains unclear whether initiating SGA adjunctive therapy earlier (after 4 weeks of treatment with SSRIs/SNRIs) can significantly improve treatment outcomes while maintaining safety in patients with an inadequate early response to SSRIs/SNRIs.

Perospirone, a novel SGA, functions as a partial agonist of the serotonin 5-HT1A receptor and an antagonist of 5-HT2A and dopamine D2 receptors.^17^ Initially approved in Japan for the treatment of schizophrenia,^18^ perospirone has demonstrated therapeutic effects on depressive symptoms, making it a promising adjunctive therapy for MDD. Preclinical studies have indicated that the combination of perospirone and antidepressants can significantly increase the level of dopamine in the medial prefrontal cortex (mPFC) of rats, indicating its potential antidepressive effect in humans.^19^^,^^20^ However, the current clinical evidence on the use of perospirone in MDD remains extremely limited, with only two reports available: a case report and an open-label study.^21^^,^^22^ The case report presented the first documented evidence that adjunctive perospirone therapy led to an improvement in depressive symptoms in a patient with treatment-resistant depression and comorbid obsessive-compulsive disorder.^21^ Subsequently, an open-label study demonstrated the effectiveness of perospirone as an adjunctive agent to antidepressants in patients with treatment-resistant depression.^22^ Regarding safety and tolerability, only one study reported that patients with MDD receiving perospirone experienced mild psychic (e.g., sleepiness, asthenia) and extrapyramidal side effects, which were generally well tolerated, with no treatment discontinuations due to adverse events (AEs).^22^ Given the limited evidence in MDD, data from other psychiatric populations may provide insights into the overall safety profile. In patients with schizophrenia, the safety profile of perospirone has been confirmed by a meta-analysis.^23^ Compared with other antipsychotics such as haloperidol, aripiprazole, and risperidone, perospirone did not increase the risk of inducing extrapyramidal symptoms (EPS), insomnia, or other common antipsychotic-related AEs; on the contrary, it was associated with fewer EPS. In summary, as an adjunctive therapeutic agent, perospirone represents a potential option for patients with MDD who exhibit inadequate response to SSRIs or SNRIs. However, the current evidence remains limited due to methodological constraints and the relatively small scale of existing studies. Well-designed, large-scale, randomized controlled trials are therefore warranted.

To determine whether perospirone is effective and safe for patients with inadequate response to SSRIs/SNRIs, we conducted a randomized, double-blind, placebo-controlled trial. The study aimed to assess whether 8 weeks of SGA adjunctive therapy can improve treatment outcomes in patients with moderate-to-severe MDD (defined as a baseline Montgomery-Åsberg Depression Rating Scale [MADRS] score of ≥20) who have exhibited an inadequate response to at least 4 weeks of treatment with SSRIs or SNRIs, and to evaluate the efficacy, safety and tolerability of perospirone as an adjunct treatment for this population.

## Methods

### Study design and participants

This study was a multi-center, two-arm, randomized, double-blind, placebo-controlled trial, with the study treatment lasting for 8 weeks. Patients were enrolled from 10 hospitals located in 10 provinces across China (see Supplementary Material 1) from December 16, 2021 to June 19, 2024. Details of the protocol are provided in the Supplementary Material 1.

All the participants were 18-to-60-year-old inpatients or outpatients from different centers. All of them met the criteria for a current episode of MDD in the Diagnostic and Statistical Manual of Mental Disorders–fifth edition (DSM-5), and their diagnosis was confirmed using the Mini International Neuropsychiatric Interview (MINI).^24^ Key inclusion criteria were (1) a MADRS score of ≥20 in the current episode,^25^ (2) inadequate response (<50% reduction in the MADRS total score) to at least one antidepressant with adequate dose and duration (≥4 weeks), and (3) having received one SSRI or SNRI at a stable dose for at least 4 weeks before screening. Patients were excluded if they were diagnosed with personality disorders, substance abuse disorders, psychotic disorders, or any mental illness other than MDD at present or in the lifetime (except for generalized anxiety disorder secondary to depression). Patients at a great risk of suicidal ideation (the score of Item 10 in MADRS was ≥4) or attempts, as determined by the clinicians, were also excluded. In addition, patients were excluded if they were using adjunctive antidepressants (i.e., concomitant use of two or more antidepressants) or antipsychotics during the current episode, had received non-pharmacological treatments (including electroconvulsive therapy, repetitive transcranial magnetic stimulation, and psychotherapy) in the last six months, had a present or past history of neurological disorders or head injury, or other major medical conditions, were pregnant or breast-feeding, or were receiving hormone therapy. The inclusion and exclusion criteria are presented in the Supplementary Material 1.

### Ethics statement

This study was performed in accordance with the Declaration of Helsinki, the International Council for Harmonization (ICH) Good Clinical Practice guidelines, the Provisions for Drug Registration, the E8 General Considerations for Clinical Trials and other regulations. The trial protocol was approved by the ethics committee of each participating center. The approval numbers from the lead center (The Second Xiangya Hospital) were (2020) EC [CR] No.130 (initial approval) and (2022) EC [CR] No.90 (amendment approval). All the participants provided written informed consent. The trial was prospectively registered on www.chictr.org.cn (ChiCTR2200063354).

### Randomization and masking

After the baseline screening, the patients were randomized at a 1:1 ratio to the perospirone group (receiving perospirone adjunctive therapy of antidepressants for 8 weeks) or the placebo group (receiving placebo and antidepressants for 8 weeks) using a computer-generated random number table. Randomization was performed using a stratified block method, with stratification based on study center. No additional stratification factors (such as sex or the class of antidepressants) were incorporated. A blinding letter was composed for the storage of the treatment code, allocation status, and treatment regimen of each participant, and the letter was not accessible to the investigators, clinicians, study personnel, or participants. Patients in the perospirone group were given perospirone hydrochloride (4–48 mg/day) in combination with their prescribed antidepressant for 8 weeks, and those in the placebo group were given placebo and their prescribed antidepressant. All the participants were assessed by experienced psychiatrists at 4 weeks, and 8 weeks after randomization.

### Treatment

During the phase of study design, the dose and administration frequency of perospirone were determined based on the approved label for schizophrenia. Participants received 8 weeks of treatment with perospirone or placebo (4 mg tablets, administered orally three times daily after food intake), while continuing their existing antidepressant treatment. The placebo tablets were identical to perospirone in terms of appearance, odor, size, and packaging, and were manufactured by the same company. Perospirone was initiated at 4 mg three times daily, with subsequent titration to a maintenance dose of 12–48 mg/day based on patient tolerance.

However, in clinical practice, dosing was pragmatically tailored by experienced clinicians based on individual patient characteristics. Treatment was generally initiated at a dosage of 4–8 mg/day, although some patients, such as those with milder symptoms or older age, initiated therapy at a reduced dose of <4 mg/day. Doses were adjusted during the first two weeks based on symptom severity and individual tolerability, and were usually stabilized by week 2. Perospirone was administered once or twice daily, with standard daily doses of 4–24 mg. For patients with more severe symptoms, higher doses (>24 mg/day) were considered on an individual basis, while remaining within the approved maximum limit of 48 mg/day.

Given that the study enrolled patients who had shown an inadequate response to at least 4 weeks of antidepressant therapy (SSRIs/SNRIs), the timing for initiating perospirone adjunctive therapy was designed with flexibility to reflect real-world clinical practice. The primary intervention timepoint was set at week 4, with treatment initiation permitted up to week 8. The SSRI/SNRI was still maintained at a stable dose throughout the 8 weeks of treatment. The use of other psychotropic medications and more than one antidepressant was prohibited, except benzodiazepine at a low dose (≤2 mg of lorazepam equivalent).

### Assessment of efficacy

The primary efficacy endpoints were the response rate (defined by an improvement in the MADRS score of ≥50% from baseline) and the remission rate (defined by a MADRS score of ≤10) at the end of week 8.^26^ The response and remission rates at the end of week 4 were also evaluated as the key secondary efficacy endpoints, which reflected the early effect of perospirone. The other key secondary efficacy endpoint was the improvement of depressive symptoms, evaluated by the changes in scores of MADRS and the 16-item Quick Inventory of Depressive Symptomatology (Self-Report) (QIDS-SR16) at the end of week 4 and week 8 from baseline. Other secondary efficacy endpoints included the improvement of anxiety symptoms, reflected by the changes in the Hamilton Anxiety Scale (HAMA) score at the end of week 4 and week 8 from baseline, and the improvement in the quality of life, reflected by the changes in the score of the Quality of Life Enjoyment and Satisfaction Questionnaire–Short Form (Q-LES-Q-SF) at the end of week 4 and week 8 from baseline.

All the investigators from different centers were given standardized training on the use of all assessment scales to ensure consistency throughout the study. The assessment using each particular scale was performed by one investigator on all patients to minimize inconsistency. Periodic quality control was also implemented.

### Safety and tolerability

Safety assessments were primarily based on patient self-reports, supplemented by the Treatment Emergent Symptom Scale (TESS) for the assessment of AEs. Serious adverse events (SAEs), defined as those resulting in hospitalization, prolonged hospital stays or life-threatening risk, were also reported. All AEs, regardless of their relationship with the investigational drug, were systematically documented. The severity of AEs was graded using the National Cancer Institute (NCI) Common Terminology Criteria for Adverse Events (CTCAE) (version 5.0), which classifies AEs into five levels of severity (Grade 1 to Grade 5).^27^ Tolerability was assessed based on the incidence of AEs that resulted in treatment discontinuation.

### Statistical analysis

Previous studies on SGA adjunctive therapy for patients with MDD indicated that the response rate of patients given SGA ranged from 10.5% to 58.9% while the response rate of those given placebo ranged from 6.8% to 46.3%.^28^ The pair of groups showing the greatest difference in response rates (46.6% vs. 26.6%) were selected as the basis for sample size calculation.^29^ A Chi-square test was performed using Power Analysis and Sample Size (PASS) version 21.0.3, which indicated that a sample size of 176 participants (88 per group) would provide 80% power (β = 0.2) at a two-sided alpha level of 0.05 (α = 0.05) to detect a significant inter-group difference in the primary efficacy outcome. Considering a potential dropout rate of 10%, the initial sample size was adjusted to 196 participants (98 per group). Given the possibility of a higher-than-expected dropout rate, the final sample size was further increased to 210 participants (105 per group), which also provides sufficient power for the remission rate (36.8% vs 18.9%) under the same assumptions.

Efficacy analyses were performed using the modified intention-to-treat analysis set (also known as the full analysis set, FAS), which included all randomized participants who received treatment and had completed at least two post-baseline efficacy assessments. For the primary efficacy endpoints (i.e., the response and remission rates at the end of week 8), missing MADRS scores in the FAS were imputed using the last observation carried forward (LOCF) method. The response and remission statuses were then determined based on the imputed MADRS scores. A logistic regression model was used to compare the outcomes between the perospirone group and the placebo group, with the placebo group being the reference category. In the logistic regression model, the treatment with perospirone was used as a factor, and the total score of MADRS at baseline was used as a covariate. As the Breslow–Day test indicated no significant heterogeneity in treatment effects across centers (p > 0.05), centers were not included as stratification factors or covariates in the final model. The assumptions of logistic regression were evaluated, and diagnostic methods and results are provided in the Supplementary Methods and Supplementary Table S1.

For the secondary efficacy endpoints, changes in MADRS, QIDS-SR16, HAMA, and Q-LES-Q-SF scores from baseline (week 0) to weeks 4 and 8 were analyzed and compared between the perospirone group and the placebo group using the mixed model for repeated measures (MMRM) analysis with an unstructured covariance matrix. The model assumed a Gaussian distribution with an identity link function. In each model, the total scores of each scales at baseline, study center (center 1 to center 10), treatment (perospirone and placebo), time (week 4 and week 8), and time × treatment interaction were included as fixed effects, and the subject-specific effect was used as a random effect. The least square mean (LSM) changes in scale scores of the two groups were recorded. The response and remission rates at the end of week 4 were analyzed using the statistical methods specified for the primary efficacy endpoints. Sensitivity analyses were performed to assess the robustness of the treatment effects under different assumptions regarding missing data and study population. Missing data in the FAS were addressed using multiple imputation under the missing-at-random assumption, and a per-protocol set (PPS) analysis was performed to assess the potential impact of protocol deviations.

The safety set (SS), consisting of individuals who received at least one dose of the randomized investigational drug, was used for the analysis of drug safety and tolerability, which was performed by comparing the self-reported AEs/SAEs between the two groups. Since the AEs covered by the TESS involved multiple systems, which could help to assess the drug safety more comprehensively, TESS was also used for safety evaluation. As the TESS was completed by patients at follow-up visits, patients who attended at least one follow-up visit were also included for the inter-group comparison of the incidence of AEs based on the TESS. For all items in TESS, a score of 0 indicates no experience of the corresponding AE, and any score greater than 0 indicates presence of the AE during treatment.

Chi-square test or Fisher's exact test was performed to compare the overall incidences of AEs, SAEs, and AEs leading to treatment discontinuation, as well as the incidence of each drug-related AE, between the two groups. The chi-square test was used when all the expected cell counts were at least 5; otherwise, Fisher's exact test was utilized. All statistical analyses were performed using the Statistical Analysis System (SAS) version 9.4, and statistical significance was determined by a two-sided p value of <0.05. No formal adjustment for multiple comparisons was prespecified in the statistical analysis plan.

### Role of the funding source

The investigational medicinal product and matching placebo were supplied by Livzon Pharmaceutical Group Inc. Statistical analysis was provided by Reboscience (Zhuhai) Pharmaceutical Research Co., Ltd. The funders and sponsor had no role in the design and conduct of the study; collection, management, and interpretation of the data; preparation, review, or approval of the manuscript; and decision to submit the manuscript for publication.

## Results

### Patient characteristics

**Fig. 1:**
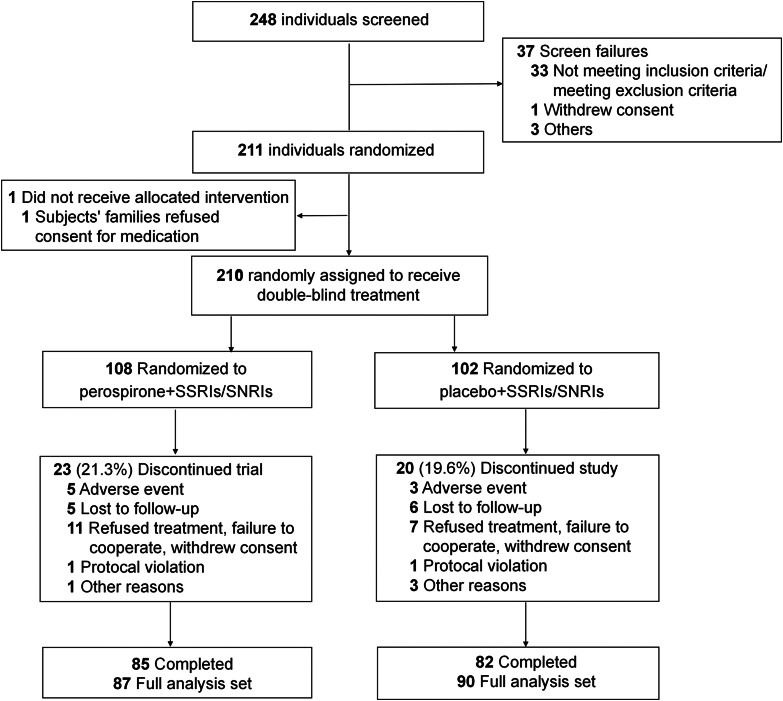
Flow diagram of participant selection. The diagram illustrates the screening, randomization, and subsequent progress of participants in the trial. SNRIs, serotonin norepinephrine reuptake inhibitors; SSRIs, selective serotonin reuptake inhibitors.

A total of 248 patients were screened between June 21, 2022 and May 15, 2024, with 37 patients failed screening for not meeting inclusion criteria or meeting any of the exclusion criteria (N = 33), withdrawal of informed consent (N = 1), and other unspecified reasons (N = 3). Of the 211 patients enrolled in the study, one patient withdrew from the trial as his family refused the administration of the study medication. Therefore, a total of 210 patients received the double-blind treatment, among whom 108 were assigned to the perospirone group and 102 to the placebo group. Finally, 85 in the perospirone group and 82 in the placebo group completed their treatment. The FAS sample consisted of a perospirone group of 87 participants and a placebo group of 90 participants, totaling 177 participants. The dropout rate was approximately 20% for both groups, for various reasons such as AEs, loss to follow-up, patient withdrawal, and violation of the protocol (Fig. 1). The SS cohort comprised 210 participants. The majority of patients received daily doses ranging from 4 to 16 mg (97.16% at week 2, 96.02% at week 4, and 95.45% at week 8), with only one patient receiving a dose >24 mg/day at week 8 (Supplementary Table S2).

The baseline demographic and clinical characteristics of the two groups were comparable and are presented in Table 1. There were no significant inter-group differences in baseline characteristics. The mean age of the study population was 29.42 ± 10.85 years, with 65.0% being female. The severity of illness was moderate to severe, with a mean MADRS total score of 28.96 ± 5.36 and a mean QIDS-SR16 total score of 15.07 ± 4.79. The most frequently prescribed class of baseline antidepressant therapy was SSRIs, administered to 76.8% of patients. Among the 177 participants, 75.14% (133/177) received 4 weeks of antidepressant treatment, white 24.86% (44/177) were treated for 4–8 weeks.

**Table 1:** Demographic and clinical characteristics of participants at baseline.

	Total (N = 177)	Placebo group (n = 90)	Perospirone group (n = 87)
**Demographic characteristics**			
Age (years)			
Mean ± SD	29.42 ± 10.85	29.31 ± 11.05	29.53 ± 10.69
Median (range)	26.00 (18.00–60.00)	24.00 (18.00–57.00)	27.00 (18.00–60.00)
Sex, no.(%)			
Female	115 (65.0)	62 (68.9)	53 (60.9)
Education (years)			
Mean ± SD	14.11 ± 2.95	13.82 ± 3.14	14.43 ± 2.70
Median (range)	15.00 (6.00–24.00)	15.00 (6.00–21.00)	15.00 (8.00–24.00)
**Psychiatric history**			
Age of first episode			
Mean ± SD	25.17 ± 10.93	25.35 ± 10.68	24.99 ± 11.27
Total duration of illness (months)			
Mean ± SD	46.10 ± 48.75	44.74 ± 46.69	47.52 ± 51.09
Frequency of episodes			
Single episode, no.(%)	20 (11.30)	7 (7.78)	13 (14.94)
Recurrent, no.(%)	43 (24.29)	22 (24.44)	21 (24.14)
Unknown, no.(%)	114 (64.41)	61 (67.78)	53 (60.92)
Antidepressant therapy			
SSRIs, no.(%)	136 (76.84)	65 (72.22)	71 (81.61)
SNRIs, no.(%)	41 (23.16)	25 (27.78)	16 (18.39)
Duration of antidepressant treatment			
4 weeks, no.(%)	133 (75.14)	68 (75.56)	65 (74.71)
More than 4 weeks, no.(%)	44 (24.86)	22 (24.45)	22 (25.29)
**Baseline severity of disease**			
MADRS score			
Mean ± SD	28.96 ± 5.36	29.06 ± 5.17	28.86 ± 5.58
Moderate:20–30, no.(%)	111 (62.71)	57 (63.33)	54 (62.07)
Severe: ≥31, no.(%)	66 (37.29)	33 (36.67)	33 (37.93)
QIDS-SR16 score			
Mean ± SD	15.07 ± 4.79	15.00 ± 4.51	15.14 ± 5.08
HAMA score			
Mean ± SD	19.52 ± 7.31	19.66 ± 7.09	19.38 ± 7.57
Q-LES-Q-SF score			
Mean ± SD	38.34 ± 8.66	38.08 ± 8.45	38.62 ± 8.91

Abbreviations: HAMA, Hamilton Anxiety Scale; MADRS, Montgomery-Åsberg Depression Rating Scale; QIDS-SR16, 16-item Quick Inventory of Depressive Symptomatology (Self-Report); Q-LES-Q-SF, Quality of Life Enjoyment and Satisfaction Questionnaire–Short Form; SD, standard deviation.

SNRI, serotonin-norepinephrine reuptake inhibitor; SSRI, selective serotonin reuptake inhibitor.

### Primary efficacy endpoints

**Fig. 2:**
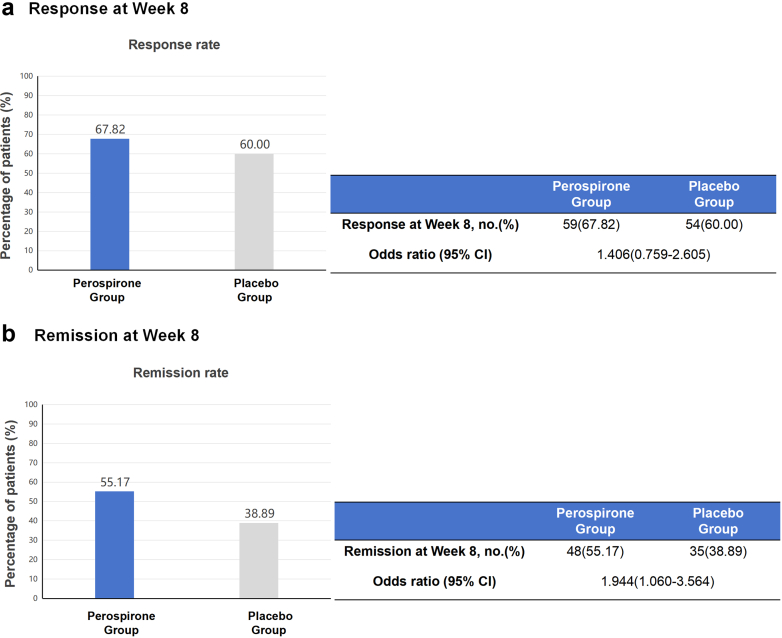
Results on the primary efficacy endpoints. (a) Response rates in the perospirone and placebo groups at week 8; (b) Remission rates in the perospirone and placebo groups at week 8.

The results of primary efficacy outcomes of the FAS sample are presented in Fig. 2. At the end of week 8, 67.8% of patients in the perospirone group and 60.0% in the placebo group were responsive. The inter-group difference was not significant, with an adjusted odds ratio (OR) of 1.406 (95% confidence interval [CI]: 0.759–2.605). However, remission occurred in more patients in the perospirone group than in the placebo group (48 patients [55.2%] vs. 35 patients [38.9%], p = 0.032, adjusted OR = 1.944, 95% CI: 1.060–3.564) at the end of week 8. Sensitivity analyses demonstrated treatment effects both directionally consistent and of comparable magnitude to those observed in the primary analysis, thereby enhancing the robustness of the observed benefit (Supplementary Tables S3–S5).

### Key secondary efficacy endpoints

The results of key secondary efficacy endpoints are presented in Table 2 and Supplementary Figure S1. At the end of week 4, a significantly higher proportion of patients in the perospirone group than in the placebo group met the priori response and remission criteria (response: 55.2% vs. 35.6%, p = 0.0090, adjusted OR = 2.243, 95% CI: 1.225–4.107; remission: 40.2% vs. 20.0%, p = 0.0040, adjusted OR = 2.701, 95% CI: 1.373–5.313).

**Table 2:** Results on the key secondary efficacy end points and other secondary efficacy end points at the end of week 4 and week 8.

	Week 4			p value	Week 8			p value
	Perospirone group (n = 87)	Placebo group (n = 90)	LSMD, vs. placebo group (95% CI)		Perospirone group (n = 87)	Placebo group (n = 90)	LSMD, vs. placebo group (95% CI)	
	LS mean (SE)	LS mean (SE)			LS mean (SE)	LS mean (SE)		
**Key secondary efficacy end points**								
MADRS score	−14.63 (0.87)	−10.26 (0.87)	−4.37 (−6.65, −2.09)	0.00020	−17.29 (0.90)	−15.46 (0.90)	−1.83 (−4.20, 0.54)	0.13
QIDS-SR16 score	−6.78 (0.51)	−4.13 (0.51)	−2.65 (−3.98, −1.32)	0.00010	−6.75 (0.59)	−5.89 (0.58)	−0.85 (−2.40, 0.69)	0.28
**Other secondary efficacy end points**								
HAMA score	−8.71 (0.73)	−6.54 (0.74)	−2.16 (−4.08, −0.25)	0.027	−10.62 (0.70)	−8.77 (0.70)	−1.86 (−3.68, −0.03)	0.046
Q-LES-Q-SF score	4.97 (0.88)	2.33 (0.89)	2.64 (0.39, 4.89)	0.022	6.96 (1.02)	4.79 (1.03)	2.17 (−0.51, 4.85)	0.11

Abbreviations: HAMA, Hamilton Anxiety Scale; LS, least squares; LSMD, least squares mean difference; MADRS, Montgomery-Åsberg Depression Rating Scale; QIDS-SR16, 16-item Quick Inventory of Depressive Symptomatology (Self-Report); Q-LES-Q-SF, Quality of Life Enjoyment and Satisfaction Questionnaire–Short Form; SE, standard error.

The LSM (SD) change in the MADRS total score at the end of week 4 from baseline was −14.63 (0.87) in the perospirone group and −10.26 (0.87) in the placebo group. The inter-group difference in the LSM change between the two groups was significant according to the MMRM analysis (least square mean difference [LSMD] = −4.37, 95% CI: −6.65 to −2.09, p = 0.00020). However, no significant difference was observed in the 8-week LSM change between the two groups (LSMD = −1.83, 95% CI: −4.20 to 0.54, p = 0.13). Regarding self-reported depressive symptoms, a significantly greater improvement in the QIDS-SR16 score was observed in the perospirone group than in the placebo group at the end of week 4 (LSMD = −2.65, 95% CI: −3.98 to −1.32, p = 0.00010). The LSM change at the end of week 4 was −6.78 (0.51) in the perospirone group and −4.31 (0.51) in the placebo group. In line with the results measured by MADRS, the inter-group difference (based on QIDS-SR16) became insignificant at the end of week 8. The LSM change in the QIDS-SR16 total score at the end of week 8 from baseline was −6.75 (0.59) in the perospirone group and −5.89 (0.58) in the placebo group (LSMD = −0.85, 95% CI: −2.40 to 0.69, p = 0.28).

### Other secondary efficacy endpoints

The results of other secondary efficacy endpoints are presented in Table 2. Compared with placebo, the use of perospirone was associated with significantly greater improvement in anxiety symptoms at the end of week 4 and week 8. The LSM change in the HAMA total score at the end of week 4 from baseline was −8.71 (0.73) in the perospirone group and −6.54 (0.74) in the placebo group (LSMD = −2.16, 95% CI: −4.08 to −0.25, p = 0.027), and the LSM change in the HAMA total score at the end of week 8 from baseline was −10.62 (0.70) in the perospirone group and −8.77 (0.70) in the placebo group (LSMD = −1.86, 95% CI: −3.68 to −0.03, p = 0.046).

Regarding the improvement of life quality, the perospirone group showed a significantly greater increase in the score of Q-LES-Q-SF than the placebo group at the end of week 4 (4.97 vs. 2.33, LSMD = 2.64, 95% CI: 0.39–4.89, p = 0.022). However, the difference observed in week 8 only showed a trend toward a greater improvement with regard to perospirone treatment (LSMD = 2.17, 95% CI: −0.51 to 4.85, p = 0.11). All sensitivity analyses for the secondary efficacy outcomes showed effects both directionally consistent and of similar magnitude as those in the primary analysis (Supplementary Tables S6–S8).

### Safety and tolerability

Overall, 8 weeks of adjunctive perospirone treatment was safe and well-tolerated. During the treatment, 51 patients (47.2%) in the perospirone group and 41 (40.2%) in the placebo group reported AEs (Table 3), with the inter-group difference in the incidence of AEs being not statistically significant (p = 0.31). The majority of AEs were mild to moderate in severity (Grade 1–2), accounting for over 90% of all reported events in both groups, and were characterized by short duration and rapid resolution (Supplementary Table S9). Three patients (2.8%) in the perospirone group and 4 (4.0%) in the placebo group experienced SAEs, including moderately severe depressive episode with psychotic symptoms (n = 1), chest pain (n = 1), depressive episode (n = 3), and suicidal behaviors (n = 2). The proportion of patients who withdrew from the study due to any AEs was low and comparable between the two groups (4.5% vs. 3.2%, p = 0.82).

**Table 3:** Common adverse events during the treatment period.

	Perospirone group (N = 108)	Placebo group (N = 102)	p value
	Number of patients (%)		
**AEs reported by patients**			
At least one AE	51 (47.22)	41 (40.20)	
SAE(s)	3 (2.78)	4 (3.92)	0.94
AE(s) leading to discontinuation of the study drug	6 (4.51)	4 (3.20)	0.82
TEAEs occurring in ≥5% of patients in any group			
Somnolence	8 (7.41)	10 (9.80)	0.54
Dizziness	11 (10.19)	4 (3.92)	0.078

	Perospirone group (N = 99)	Placebo group (N = 96)	p value
	Number of patients (%)		
**AEs assessed by TESS**			
Dry month	41 (41.41)	45 (46.55)	0.44
Somnolence	40 (40.40)	40 (41.67)	0.86
Insomnia	30 (30.30)	36 (37.50)	0.29

Abbreviations: AEs, adverse events; SAEs, severe adverse events; TESS, Treatment Emergent Symptom Scale.

The incidences of common (with an incidence of 5% or higher) AEs reported by patients are presented in Table 3. The common AEs in the perospirone group were dizziness (n = 11, 10.19%) and somnolence (n = 8, 7.41%). The inter-group comparison of the incidences of AEs based on TESS is presented in Table 3 (only the top three AEs with the highest incidences are shown). The most common AEs in patients treated with perospirone were dry mouth (n = 41, 41.4%), somnolence (n = 40, 40.4%), and insomnia (n = 30, 30.3%). However, no significant differences in the incidences of any AEs reported by patients or identified using TESS were found between the two groups (all p values > 0.05). Further details on AEs are provided in Supplementary Tables S10 and S11.

### Subgroup analyses

The baseline demographic and clinical characteristics were comparable between the two groups, except that the severity of anxiety was significantly higher in the 4-to-8-week group (Supplementary Table S12). In the 4-week group, adjunctive therapy with perospirone demonstrated a trend toward improved treatment outcomes at week 8 compared to placebo (response: adjusted OR = 1.368; remission: adjusted OR = 1.488, Supplementary Table S13). Larger effect sizes were observed in the 4-to-8-week group (response: adjusted OR = 1.901, remission: adjusted OR = 4.967, Supplementary Tables S14 and S15). However, the test for the treatment–subgroup interaction did not reach statistical significance (response: p = 0.883; remission: p = 0.122), as determined by the logistic regression analysis (Supplementary Methods and Supplementary Table S16).

Additionally, in the 4-week group, adjunctive perospirone demonstrated a favorable trend compared to placebo across multiple measures, including improvements in MADRS, HAMA, QIDS-SR16, and Q-LES-Q-SF scores, though the magnitude of changes was smaller than that in the 4-to-8-week group (Supplementary Tables S17–S19).

## Discussion

To our knowledge, this is the first multi-center, randomized, double-blind, placebo-controlled trial to evaluate the efficacy and safety of perospirone as an adjunctive therapy in patients with MDD who failed to achieve an adequate response to at least one antidepressant for at least 4 weeks during the current episode. The study showed that (1) early SGA adjunctive therapy had the potential to improve treatment outcomes in patients who exhibited inadequate responses to conventional antidepressants at the early stage of treatment; (2) 8 weeks of treatment with perospirone as an adjunct agent to standard antidepressant treatment exhibited a trend toward higher response and remission rates in this patient population; (3) 8 weeks of treatment with perospirone at a dose of 4–16 mg/day was safe and well tolerated.

Consistent with guidelines (CANMAT and RANZCP) and broader evidence, early adjustment of treatment regimens improved the outcomes of patients with an inadequate response to antidepressants.^4^^,^^8^^,^^30^, ^31^, ^32^ In the present study, numerically higher response and remission rates were observed in the perospirone group at week 8 (response: 67.8% vs. 60.0%; remission: 55.2% vs. 38.9%), with a nominally significant difference in the remission rate (p = 0.032) but not in the response rate (p = 0.28). The high placebo response rate (>40.0%) may have contributed to the attenuated drug-placebo difference, as evidenced by previous studies on MDD.^33^^,^^34^ This may be attributed to the inclusion of delayed responders, who exhibited inadequate early improvement yet demonstrated progressive improvement over time in the absence of active intervention. Additionally, the high incidence of AEs in the placebo group (40.2%) might have prompted patients to perceive themselves as receiving active treatment, regardless of the actual treatment allocation; this expectancy effect could have potentiated the placebo response.^35^^,^^36^ Therefore, the lack of significant benefit may not necessarily indicate therapeutic inefficacy, but could instead reflect an overestimation of the placebo effect.

Notably, adjunctive perospirone significantly improved the response and remission rates after 4 weeks of treatment (adjusted OR = 2.243, p = 0.0090 and adjusted OR = 2.701, p = 0.0040, respectively), with both improvements remaining statistically significant under a more conservative significance threshold (p < 0.025). Sensitivity analyses consistently corroborated the findings, thereby reinforcing their robustness. Furthermore, adjunctive perospirone was associated with greater improvements than placebo in MADRS, QIDS-SR16, HAMA and Q-LES-Q-SF scores at week 4, indicating both rapid symptom relief and early improvement in quality of life. These findings support the consideration of a 4-week adjunctive perospirone regimen for patients with MDD who exhibit inadequate early response and more severe symptoms, particularly those requiring rapid clinical improvement.

Given that 25% of inlcuded participants had received longer antidepressant treatment (4–8 weeks) before randomization, subgroup analyses were conducted to specifically evaluate the efficacy of adjunctive perospirone in patients with inadequate response who had only received 4 weeks of treatment. The results suggested a trend toward improved response (adjusted OR = 1.368) and remission rates (adjusted OR = 1.488) with adjunctive perospirone in the 4-week group, indicating potential benefits at an early stage. Although the observed effect sizes were smaller than those in patients with longer durations of inadequate response (4–8 weeks), the interactions between treatment and subgroup were not statistically significant for either primary outcomes. Early symptom improvement is a well-established predictor of subsequent remission and functional recovery in social and cognitive domains, and is also associated with greater patient confidence in the treatment and better adherence.^8^^,^^37^^,^^38^ Therefore, even modest early benefits may justify timely treatment optimization rather than deferring intervention until more pronounced effects emerge. Initiating adjunctive SGAs such as perospirone early in patients with inadequate response within the first 4 weeks may represent a proactive strategy to improve long-term outcomes. However, these analyses were conducted post hoc and should therefore be interpreted as exploratory.

In addition, adjunctive perospirone was associated with significant improvement in anxiety symptoms in the studied population. Preclinical studies have found anxiolytic-like effects of perospirone in rats,^39^^,^^40^ while several clinical case reports have documented the effects of perospirone in alleviating anxiety symptoms among patients with schizophrenia or dissociative identity disorder.^41^^,^^42^ These findings suggest that perospirone may have intrinsic anxiolytic properties. The observed therapeutic benefits may be attributed to its pharmacological properties, especially its 5-HT1A partial agonism and 5-HT2A antagonism–mechanisms that have been independently linked to anxiolytic effects in prior studies.^43^, ^44^, ^45^, ^46^ However, the anxiolytic effect of perospirone in patients with MDD remains insufficiently investigated, and the present study represents the first evidence indicating such effect. Further research is warranted to elucidate whether this anxiolytic effect is mediated directly through the action on 5-HT receptors or indirectly through the improvement in depressive symptoms.

The adjunctive use of perospirone was safe and well tolerated among participants in the present study. A prior systematic review of phase II and III clinical trials involving Japanese patients with schizophrenia reported that common AEs during treatment with perospirone included extrapyramidal symptoms (e.g., akathisia, tremor, and muscular rigidity) and insomnia.^18^ In the present study, the common AEs observed in the perospirone group primarily involved arousal-related symptoms (somnolence and insomnia), autonomic nervous system disorders (e.g., dry mouth, constipation, and sweating), and extrapyramidal symptoms (tremor and akathisia), with the highest incidence being 46%.

Although the incidence of AEs was relatively high in the perospirone group (47.2%), this did not differ significantly from the placebo group. The majority of AEs (97.0%) were mild to moderate and transient, with 81.2% resolving during the study period. While most AEs in the perospirone group were considered “possibly” or “probably” related to the treatment, the causality pattern did not differ significantly from that in the placebo group (p = 0.058). These similarities suggest that many of the reported AEs may not be specifically attributable to perospirone but could instead reflect manifestations of the underlying depression, comorbid anxiety, or concomitant antidepressant use. Notably, symptoms such as restlessness and sweating are common in anxiety disorders and may be misattributed to treatment. Furthermore, the high incidence of AEs in both groups may also result from nocebo effects and heightened symptom awareness in the clinical trial setting.^47^^,^^48^ Collectively, the present study demonstrated that 8 weeks of treatment with perospirone at a dose of 4–16 mg/day is safe and tolerable for the studied population.

Several limitations should be taken into account when interpreting the results. First, this study evaluated two primary outcomes without multiplicity adjustments, which might have increased the risk of false positivity. Second, blinding integrity was not formally assessed in this study. The high incidence of AEs in the placebo group may lead to functional unblinding and heightened expectations of patients, potentially amplifying the placebo effect and thereby confounding the results. Third, the dropout rate (approximately 20%) exceeded the anticipated 10%, which might have reduced the statistical power due to a smaller-than-expected effective sample size. Fourth, the placebo group had a relatively high response rate (60.0%). Our study included patients with inadequate response to SSRIs/SNRIs after at least 4 weeks of treatment, which might have resulted in the inclusion of patients who were delayed responders rather than true non-responders to SSRIs/SNRIs. Fifth, the history of prior antidepressant treatment was not systematically collected, which might have led to heterogeneity among participants. Sixth, perospirone was administered within an individually titrated dose range of 4–24 mg/day, rather than as a fixed regimen, which might have introduced some variability in treatment response. However, this approach reflects real-world clinical practice and improves tolerability and adherence. Finally, without a delayed-initiation comparator arm, this study could not directly compare the effects of treatment timings, such as initiating therapy at 4 weeks vs. 6–8 weeks. Therefore, future prospective, fixed-dose, head-to-head randomized controlled trials are needed to assess the efficacy of different timing strategies for the adjunctive therapy.

In summary, despite the lack of clear evidence supporting significant improvement in the primary outcome, the observed trend toward improved treatment outcomes and the significant rapid-onset effect suggest that early adjunctive perospirone (initiated after 4 weeks of treatment with SSRIs/SNRIs) may be a viable option for patients with MDD who exhibit inadequate response to antidepressants, particularly those with more severe symptoms. Further large-scale, long-term follow-up studies are warranted to validate these findings and evaluate the long-term benefit and safety of perospirone in the maintenance therapy.

## Contributors

Drs L. Li, Yan Zhang and J. Liu accessed and verified the data, had full access to all of the data in the study, and take responsibility for the integrity of the data and the accuracy of the data analysis.

Concept and design: L. Li, Yan Zhang, J. Liu, Gao. Acquisition, analysis, or interpretation of data: J. Liu, Gao, Ju, L. Zhang, Z. Li, Yan, Z. Wang, Zhou, Q. Zhang, Zhao. Drafting of the manuscript: J. Liu, Gao, Liao, Zheng, Cai. Critical review of the manuscript for important intellectual content: L. Li, Yan Zhang, Yao, Kuang, Yang, Hu. Administrative, technical, or material support: B. Wang, Ding, Yu, Yunshu Zhang, K. Li, G. Liu, Wei, Zheng, B. Liu, Liao, Tang, Jiang, Hu. Supervision: L. Li, Yan Zhang.

## Data sharing statement

Deidentified participant data from this study will be made available upon publication of the associated article. Approved researchers may request access for legitimate academic purposes by submitting a methodological proposal to the corresponding authors. Approved requests will require execution of a data access agreement. Additional details regarding data access procedures will be provided upon publication. Researchers could contact the corresponding authors (Yan Zhang, yan.zhang@csu.edu.cn; Lingjiang Li, LLJ2920@csu.edu.cn) for all data inquiries.

## Declaration of interests

We declare no competing interests. The funders and sponsor had no role in the design and conduct of the study; collection, management, and interpretation of the data; preparation, review, or approval of the manuscript; and decision to submit the manuscript for publication.

## Appendix A Supplementary data

Study Protocol

## References

[1] Nelson J.C., Papakostas G.I. (2009). Atypical antipsychotic augmentation in major depressive disorder: a meta-analysis of placebo-controlled randomized trials. Am J Psychiatry.

[2] Spielmans G.I., Berman M.I., Linardatos E. (2013). Adjunctive atypical antipsychotic treatment for major depressive disorder: a meta-analysis of depression, quality of life, and safety outcomes. PLoS Med.

[3] Bai B., Li Y., Chen X. (2025). The augmentative efficacy of second-generation anti-psychotics (SGA) to anti-depressants in treating treatment-resistant depression: a network meta-regression analysis. BMC Psychiatry.

[4] Malhi G.S., Bell E., Singh A.B. (2020). The 2020 royal Australian and New Zealand College of Psychiatrists clinical practice guidelines for mood disorders: major depression summary. Bipolar Disord.

[5] Lam R.W., Kennedy S.H., Adams C. (2024). Canadian Network for Mood and Anxiety Treatments (CANMAT) 2023 Update on Clinical Guidelines for Management of Major Depressive Disorder in Adults: réseau canadien pour les traitements de l’humeur et de l’anxiété (CANMAT) 2023 : mise à jour des lignes directrices cliniques pour la prise en charge du trouble dépressif majeur chez les adultes. Can J Psychiatry Rev Can Psychiatr.

[6] Henssler J., Kurschus M., Franklin J. (2018). Trajectories of acute antidepressant efficacy: how long to wait for response? A systematic review and meta-analysis of long-term, placebo-controlled acute treatment trials. J Clin Psychiatry.

[7] Nierenberg A.A., McLean N.E., Alpert J.E. (1995). Early nonresponse to fluoxetine as a predictor of poor 8-week outcome. Am J Psychiatry.

[8] Oluboka O.J., Katzman M.A., Habert J. (2018). Functional recovery in major depressive disorder: providing early optimal treatment for the individual patient. Int J Neuropsychopharmacol.

[9] Baune B.T., Falkai P. (2021). Changes in antidepressant therapy should be considered early in patients with inadequate response to a first-line agent. Aust N Z J Psychiatry.

[10] Henkel V., Seemüller F., Obermeier M. (2009). Does early improvement triggered by antidepressants predict response/remission? — analysis of data from a naturalistic study on a large sample of inpatients with major depression. J Affect Disord.

[11] Baldwin D.S., Stein D.J., Dolberg O.T. (2009). How long should a trial of escitalopram treatment be in patients with major depressive disorder, generalised anxiety disorder or social anxiety disorder? An exploration of the randomised controlled trial database. Hum Psychopharmacol.

[12] Kudlow P.A., McIntyre R.S., Lam R.W. (2014). Early switching strategies in antidepressant non-responders: current evidence and future research directions. CNS Drugs.

[13] Fava M., Mischoulon D., Iosifescu D. (2012). A double-blind, placebo-controlled study of aripiprazole adjunctive to antidepressant therapy among depressed outpatients with inadequate response to prior antidepressant therapy (ADAPT-A study). Psychother Psychosom.

[14] El-Khalili N., Joyce M., Atkinson S. (2010). Extended-release quetiapine fumarate (quetiapine XR) as adjunctive therapy in major depressive disorder (MDD) in patients with an inadequate response to ongoing antidepressant treatment: a multicentre, randomized, double-blind, placebo-controlled study. Int J Neuropsychopharmacol.

[15] Thase M.E., Youakim J.M., Skuban A. (2015). Adjunctive brexpiprazole 1 and 3 mg for patients with major depressive disorder following inadequate response to antidepressants: a phase 3, randomized, double-blind study. J Clin Psychiatry.

[16] Papakostas G.I., Fava M., Baer L. (2015). Ziprasidone augmentation of escitalopram for major depressive disorder: efficacy results from a randomized, double-blind, placebo-controlled study. Am J Psychiatry.

[17] Kato T., Hirose A., Ohno Y. (1990). Binding profile of SM-9018, a novel antipsychotic candidate. Jpn J Pharmacol.

[18] Onrust S.V., McClellan K. (2001). Perospirone. CNS Drugs.

[19] Morita M., Nakayama K. (2011). Mirtazapine in combination with perospirone synergistically enhances dopamine release in the rat prefrontal cortex via 5-HT1A receptor activation. Psychiatry Clin Neurosci.

[20] Yoshino T., Nisijima K., Shioda K. (2004). Perospirone, a novel atypical antipsychotic drug, potentiates fluoxetine-induced increases in dopamine levels via multireceptor actions in the rat medial prefrontal cortex. Neurosci Lett.

[21] Otsuka T., Togo T., Sugiyama N. (2007). Perospirone augmentation of paroxetine in treatment of refractory obsessive-compulsive disorder with depression. Prog Neuropsychopharmacol Biol Psychiatry.

[22] Sato Y., Yasui-Furukori N., Nakagami T. (2009). Augmentation of antidepressants with perospirone for treatment-resistant major depressive disorder. Prog Neuropsychopharmacol Biol Psychiatry.

[23] Kishi T., Iwata N. (2013). Efficacy and tolerability of perospirone in schizophrenia: a systematic review and meta-analysis of randomized controlled trials. CNS Drugs.

[24] Sheehan D.V., Lecrubier Y., Sheehan K.H. (1998). The Mini-International Neuropsychiatric Interview (M.I.N.I.): the development and validation of a structured diagnostic psychiatric interview for DSM-IV and ICD-10. J Clin Psychiatry.

[25] Montgomery S.A. (1985). Development of new treatments for depression. J Clin Psychiatry.

[26] Montgomery S.A., Asberg M. (1979). A new depression scale designed to be sensitive to change. Br J Psychiatry.

[27] Freites-Martinez A., Santana N., Arias-Santiago S. (2021). Using the common terminology criteria for adverse events (CTCAE–Version 5.0) to evaluate the severity of adverse events of anticancer therapies. Actas Dermosifiliogr.

[28] Scott F., Hampsey E., Gnanapragasam S. (2023). Systematic review and meta-analysis of augmentation and combination treatments for early-stage treatment-resistant depression. J Psychopharmacol.

[29] Berman R.M., Fava M., Thase M.E. (2009). Aripiprazole augmentation in major depressive disorder: a double-blind, placebo-controlled study in patients with inadequate response to antidepressants. CNS Spectr.

[30] Kennedy S.H., Lam R.W., McIntyre R.S. (2016). Canadian network for mood and anxiety treatments (CANMAT) 2016 clinical guidelines for the management of adults with major depressive disorder: section 3. Pharmacological treatments. Can J Psychiatry.

[31] Habert J., Katzman M.A., Oluboka O.J. (2016). Functional recovery in major depressive disorder: focus on early optimized treatment. Prim Care Companion CNS Disord.

[32] Oluboka O.J., Habert J., Khullar A. (2025). Urgency to treat and early optimized treatment in major depressive disorder: consequences of delayed treatment, barriers to implementation, and practical strategies for clinicians. CNS Spectr.

[33] Walsh B.T., Seidman S.N., Sysko R. (2002). Placebo response in studies of major depression: variable, substantial, and growing. JAMA.

[34] Iovieno N., Papakostas G.I. (2012). Correlation between different levels of placebo response rate and clinical trial outcome in major depressive disorder: a meta-analysis. J Clin Psychiatry.

[35] Schenk L.A., Fadai T., Büchel C. (2024). How side effects can improve treatment efficacy: a randomized trial. Brain J Neurol.

[36] Huneke N.T.M., Fusetto Veronesi G., Garner M. (2025). Expectancy effects, failure of blinding integrity, and placebo response in trials of treatments for psychiatric disorders: a narrative review. JAMA Psychiatry.

[37] Kraus C., Kadriu B., Lanzenberger R. (2019). Prognosis and improved outcomes in major depression: a review. Transl Psychiatry.

[38] Sumiyoshi T., Hoshino T., Mishiro I. (2022). Prediction of residual cognitive disturbances by early response of depressive symptoms to antidepressant treatments in patients with major depressive disorder. J Affect Disord.

[39] Sakamoto H., Matsumoto K., Ohno Y. (1998). Anxiolytic-like effects of perospirone, a novel serotonin-2 and dopamine-2 antagonist (SDA)-type antipsychotic agent. Pharmacol Biochem Behav.

[40] Ishida-Tokuda K., Ohno Y., Sakamoto H. (1996). Evaluation of perospirone (SM-9018), a novel serotonin-2 and dopamine-2 receptor antagonist, and other antipsychotics in the conditioned fear stress-induced freezing behavior model in rats. Jpn J Pharmacol.

[41] Okugawa G., Nobuhara K., Kitashiro M. (2005). Perospirone for treatment of dissociative identity disorder. Psychiatry Clin Neurosci.

[42] Roppongi T., Togo T., Nakamura S. (2007). Perospirone in treatment of Huntington's disease: a first case report. Prog Neuropsychopharmacol Biol Psychiatry.

[43] Gross C., Zhuang X., Stark K. (2002). Serotonin1A receptor acts during development to establish normal anxiety-like behaviour in the adult. Nature.

[44] Hershenberg R., Gros D.F., Brawman-Mintzer O. (2014). Role of atypical antipsychotics in the treatment of generalized anxiety disorder. CNS Drugs.

[45] Taddeucci A., Olivero G., Roggeri A. (2022). Presynaptic 5-HT2A-mGlu2/3 receptor-receptor crosstalk in the prefrontal cortex: metamodulation of glutamate exocytosis. Cells.

[46] Weisstaub N.V., Zhou M., Lira A. (2006). Cortical 5-HT2A receptor signaling modulates anxiety-like behaviors in mice. Science.

[47] Colloca L. (2024). The nocebo effect. Annu Rev Pharmacol Toxicol.

[48] Phillips R., Hazell L., Sauzet O. (2019). Analysis and reporting of adverse events in randomised controlled trials: a review. BMJ Open.

