# Supplementary material for: Efficacy and safety of perospirone as adjunctive therapy in major depressive disorder patients with inadequate response to antidepressants: a randomized clinical trial
Source: eClinicalMedicine. 2025 Nov 7;90:103626. doi: 10.1016/j.eclinm.2025.103626 (PMC12766416; doi:10.1016/j.eclinm.2025.103626)
Supplement: Study Protocol [file mmc1.doc]

**Supplementary Appendix B Study Protocol**

**Efficacy and Safety of Perospirone Augmentation in Major Depressive Disorder Patients With Inadequate Response to Antidepressants: A Randomized Clinical Trial**

Register number: ChiCTR2200063354

Protocol Code: PLPL-RCT

Principal Investigator: Professor Lingjiang Li

Sponsoring and Leading Unit: The Second Xiangya Hospital of Central South University

Version 2.0/ Date: 16.12.2021

**Table of contents**

[List of abbrevations 9](#__RefHeading___Toc14788)

[Compliance Statement 11](#__RefHeading___Toc4558)

[1. Synopsis 12](#__RefHeading___Toc7116)

[1.1 Overview 12](#__RefHeading___Toc29611)

[1.2 Flowchart 16](#__RefHeading___Toc17912)

[1.3 Study schedule 18](#__RefHeading___Toc20233)

[2. Introduction 20](#__RefHeading___Toc19163)

[2.1 Background 20](#__RefHeading___Toc3462)

[2.2 Risk/Benefit Assessment 22](#__RefHeading___Toc29074)

[2.2.1 Identified Potential Risks 22](#__RefHeading___Toc5620)

[2.2.2 Identified Potential Benefits 22](#__RefHeading___Toc31016)

[2.2.3 Potential Risk/Benefit Assessment 22](#__RefHeading___Toc22707)

[3. Research Objectives and Endpoints 24](#__RefHeading___Toc28688)

[4. Study Design 27](#__RefHeading___Toc21419)

[4.1 Overall design 27](#__RefHeading___Toc2229)

[4.2 The scientific rationale of the study design 27](#__RefHeading___Toc29501)

[4.3 The rationale for drug dosage selection 27](#__RefHeading___Toc23330)

[4.4 The definition of study termination 27](#__RefHeading___Toc8108)

[4.5 Randomization method 28](#__RefHeading___Toc17745)

[4.6 Blinding, unblinding and breaking the blind operation procedures 28](#__RefHeading___Toc710)

[4.6.1 Blinding code storage 28](#__RefHeading___Toc26285)

[4.6.2 Blinding code storage 28](#__RefHeading___Toc12244)

[4.6.3 Unblinding under emergency 29](#__RefHeading___Toc22716)

[5. Study Population 30](#__RefHeading___Toc26654)

[5.1 Inclusion criteria 30](#__RefHeading___Toc5653)

[5.2 Exclusion criteria 30](#__RefHeading___Toc18570)

[5.3 Lifestyle precautions 31](#__RefHeading___Toc18957)

[5.4 Screening failure 31](#__RefHeading___Toc20756)

[5.5 Recruitment and retention strategies 31](#__RefHeading___Toc22204)

[6. Study Intervention 32](#__RefHeading___Toc32058)

[6.1 Management of study intervention 32](#__RefHeading___Toc3153)

[6.1.1 Description of study intervention 32](#__RefHeading___Toc7308)

[6.1.2 Dosing and administration 32](#__RefHeading___Toc9158)

[6.2 Management of investigational medications 32](#__RefHeading___Toc21765)

[6.2.1 Supply and dispensing of investigational medications 32](#__RefHeading___Toc24693)

[6.2.2 Formulation, appearance, packaging, and labeling of investigational medications 32](#__RefHeading___Toc17618)

[6.2.3 Storage conditions 33](#__RefHeading___Toc5920)

[6.3 Methods to reduce deviations 33](#__RefHeading___Toc10166)

[6.4 Patient compliance with study interventions 33](#__RefHeading___Toc3686)

[6.5 Concomitant medications 33](#__RefHeading___Toc17454)

[6.5.1 Permitted concomitant medications 33](#__RefHeading___Toc3008)

[6.5.2 Prohibited and cautionary concomitant medications 33](#__RefHeading___Toc18168)

[6.5.3 Emergency drugs 34](#__RefHeading___Toc14710)

[7. Termination of Study Intervention and Discontinuation/Withdrawal of Participants 35](#__RefHeading___Toc10169)

[7.1 Termination of study intervention 35](#__RefHeading___Toc13614)

[7.2 Discontinuation/withdrawal of participants 35](#__RefHeading___Toc18375)

[7.3 Lost to follow-up 35](#__RefHeading___Toc21506)

[8. Study Assessments and Procedures 37](#__RefHeading___Toc17518)

[8.1 Efficacy assessments 37](#__RefHeading___Toc20547)

[8.1.1 Primary efficacy assessments 37](#__RefHeading___Toc37)

[8.1.2 Secondary efficacy end points assessments 37](#__RefHeading___Toc30289)

[8.2 Safety assessments 38](#__RefHeading___Toc17028)

[8.3 Trial procedure 38](#__RefHeading___Toc20784)

[8.3.1 Baseline screening 38](#__RefHeading___Toc3342)

[8.3.2 Visit 1 39](#__RefHeading___Toc7360)

[8.3.3 Visit 2 39](#__RefHeading___Toc29436)

[8.3.4 Visit 3 39](#__RefHeading___Toc12995)

[8.4 Adverse event (AE) and serious adverse event (SAE) 39](#__RefHeading___Toc29869)

[8.4.1 Definition of adverse event (AE) 39](#__RefHeading___Toc4380)

[8.4.2 Definition of serious adverse event (SAE) 40](#__RefHeading___Toc9078)

[8.4.3 Severity of AEs 40](#__RefHeading___Toc19174)

[8.4.4 Management of AEs 41](#__RefHeading___Toc14544)

[8.4.5 Causality assessments 41](#__RefHeading___Toc848)

[8.4.6 Requirements for AE recording and follow-up 42](#__RefHeading___Toc25672)

[8.4.7 AE reporting 42](#__RefHeading___Toc18537)

[8.4.8 SAE reporting 42](#__RefHeading___Toc28058)

[8.4.9 Reporting AEs/SAEs to participants 43](#__RefHeading___Toc5440)

[8.4.10 Adverse event of special interest 43](#__RefHeading___Toc29905)

[8.4.11 Pregnancy reporting 43](#__RefHeading___Toc15873)

[8.5 Unanticipated problem (UP) 44](#__RefHeading___Toc10359)

[8.5.1 Definition of unanticipated problem (UP) 44](#__RefHeading___Toc24052)

[8.5.2 UPs reporting 44](#__RefHeading___Toc18955)

[8.5.3 Suspected unexpected serious adverse event (SUSAR) reporting 45](#__RefHeading___Toc24208)

[8.5.4 Reporting UPs to participants 45](#__RefHeading___Toc23381)

[9. Statistical Analysis 47](#__RefHeading___Toc25440)

[9.1 Analytical set 47](#__RefHeading___Toc20134)

[9.1.1 Modified intention-to-treat (mITT) set/full analysis set (FAS) 47](#__RefHeading___Toc28690)

[9.1.2 Safety set (SS) 47](#__RefHeading___Toc25023)

[9.2 Statistical analyse 47](#__RefHeading___Toc14384)

[9.2.1 Sample size calculation 47](#__RefHeading___Toc24793)

[9.2.2 Efficacy end points analyse 47](#__RefHeading___Toc17905)

[9.2.3 Safety end points analyses 48](#__RefHeading___Toc29474)

[9.2.4 Statistical analysis methods 48](#__RefHeading___Toc26071)

[10. Supporting Documentation and Operational Considerations 50](#__RefHeading___Toc31454)

[10.1 Regulatory, ethical, and study oversight considerations 50](#__RefHeading___Toc29253)

[10.1.1 Informed consent process 50](#__RefHeading___Toc11981)

[10.1.2 Suspension and termination of the study 50](#__RefHeading___Toc32367)

[10.1.3 Confidentiality and privacy 51](#__RefHeading___Toc30601)

[10.1.4 Future utilization of stored samples and data 51](#__RefHeading___Toc11560)

[10.1.5 Clinical trial monitoring 52](#__RefHeading___Toc24247)

[10.1.6 Quality control (QC) 52](#__RefHeading___Toc29491)

[10.1.7 Management of data and records collection and retention 53](#__RefHeading___Toc6390)

[10.1.8 Protocol violation 53](#__RefHeading___Toc8694)

[10.1.9 Publication and Data Sharing Policy 54](#__RefHeading___Toc27728)

[10.1.10 Conflict of interest policy 54](#__RefHeading___Toc8355)

[10.2 Other considerations 54](#__RefHeading___Toc16181)

[11. References 56](#__RefHeading___Toc4046)

[12. Protocol Adjustments During the Implementation of the Trial 57](#__RefHeading___Toc15429)

# List of abbrevations

| AE | Adverse Event |
| --- | --- |
| ALB | Albumin |
| ALP | Alkaline Phosphatase |
| ALT | Alanine Aminotransferase |
| ANC | Absolute Neutrophil Count |
| AST | Aspartate Aminotransferase |
| BIL | Bilirubin (in urine) |
| BLD | Blood (occult blood in urine) |
| BSI-CV | Beck Scale for Suicide Ideation - Chinese Version |
| BUN | Blood Urea Nitrogen |
| CGI | Clinical Global Impression |
| CRE | Creatinine |
| CRF | Case Report Form |
| DBIL | Direct Bilirubin |
| ECG | Electrocardiogram |
| FAS | Full Analysis Set |
| GAD-7 | Generalized Anxiety Disorder 7-item Scale |
| GLU | Glucose (in urine) |
| HAMA | Hamilton Anxiety Scale |
| HB | Hemoglobin |
| HCL-32 | Hypomania Check List |
| IBIL | Indirect Bilirubin |
| ICH GCP | International Council for Harmonisation of Technical Requirements for Pharmaceuticals for Human Use - Good Clinical Practice |
| IRB | Institutional Review Board |
| KET | Ketone Bodies (in urine) |
| LDH-L | L-Lactate Dehydrogenase |
| LYM | Lymphocyte Count |
| MADRS | Montgomery-Åsberg Depression Rating Scale |
| MDD | Major depressive disorder |
| mITT | Modified Intention-To-Treat |
| MOP | Manual of Operations Procedures |
| NIT | Nitrite (in urine) |
| NMPA | National Medical Products Administration |
| PH | pH |
| PLT | Platelet Count |
| PRO | Protein (in urine) |
| PSQI | Pittsburgh Sleep Quality Index |
| QIDS-SR16 | 16-item Quick Inventory of Depressive Symtomatology-Self-Report |
| Q-LES-Q-SF | Quality of Life Enjoyment Questionnaire - Short Form |
| RBC | Red Blood Cells |
| SAE | Serious Adverse Event |
| SDS | Sheehan Disability Scale |
| SG | Specific Gravity (of urine) |
| SGA | Second-generation antipsychotic |
| SHAPS | Snaith-Hamilton Pleasure Scale |
| SNRIs | Serotonin norepinephrine reuptake inhibitors |
| SOPs | Standard Operating Procedures |
| SSRIs | Selective serotonin reuptake inhibitors |
| TBIL | Total Bilirubin |
| TESS | Treatment-Emergent Symptom Scale |
| TP | Total Protein |
| UP | Unanticipated Problem |
| URO | Urobilinogen (in urine) |
| WBC | White Blood Cells |
| YMSR | Young Mania Rating Scale |

# Compliance Statement

This study will be conducted in accordance with the International Conference on Harmonisation (ICH) Good Clinical Practice (GCP) guidelines and the following regulations:

- Good Clinical Practice: Consolidated Guideline (July 2020)
- Drug Registration Management Measures (July 2020)
- E8: General Considerations for Clinical Trials (2007)
- ICH E10: Choice of Control Group and Related Issues in Clinical Trials (2007)
- Declaration of Helsinki (2000)
- Code of Practice for Ethical Committees in Research Institutions (Edited by Wang Xiangqian and Tang Yizhong, Science Press, 2007)

Researchers and staff responsible for conducting, managing, and supervising clinical trials must complete training related to human subject protection and GCP.

This study protocol, informed consent form, recruitment materials, and all related documents will be submitted to the Institutional Review Board (IRB) for review and approval. Prior to enrolling subjects, it is mandatory to obtain approval for the protocol and informed consent documents. All amendments to the protocol must be reviewed and approved by the IRB. Additionally, any changes to the informed consent documents must be approved by the IRB. It should also be determined whether subjects who have already signed the approved version of the informed consent document need to sign a new version.

1. Synopsis

**1.1 Overview**

| Title | Efficacy and safety of augmentation pharmacotherapy with perospirone for major depressive disorder patients resistant to antidepressants: a randomised, double-blind, placebo-controlled trial |
| --- | --- |
| Research Description | Patients with major depressive disorder (MDD) are often treated with one or more antidepressants. However, some patients do not respond adequately to treatment with at least one antidepressant at an adequate dosage and duration. Evidence-based medical evidence suggests that combining a second-generation antipsychotics (SGA) with antidepressant treatment can improve treatment outcomes for such patients. Perospirone is a novel SGA, which has been approved for the treatment of patients with schizophrenia in Japan. To evaluate the efficacy and safety of perospirone combined with antidepressant treatment for patients who are resistant to antidepressants, and to provide better guidance for clinical practice, a study titled "Efficacy and safety of augmentation pharmacotherapy with perospirone for major depressive disorder patients resistant to antidepressants: a randomised, double-blind, placebo-controlled trial" is currently underway. |
| Objective | To evaluate the efficacy, safety and tolerability of perospirone administered as an add-on to stable antidepressant medication for 8 weeks copmared with placebo in patients with MDD who had inadequate response to selective serotonin reuptake inhibitors(SSRIs) or serotonin norepinephrine reuptake inhibitors (SNRIs). |
| Study Design | Multi-centre, 2-armed parallel group, randomized, double-blind, placebo-controlled trial. The perospirone or placebo drug is used as an adjunctive medication to standard therapy consisting of conventional antidepressant medication (SSRIs/SNRIs). |
| Trial mediation/ treatment strategy | 4-48mg/d Perospirone (three times a day) |
| Comparative medication | Placebo |
| Total number of patients | 210 |
| Research centers | Beijing Huilongguan Hospital (Beijing, China), Hebei Mental Health Center (Hebei, China), Nanjing Brain Hospital (Nanjing, China), Xiamen Xianyue Hospital (Xiamen, China), The First Affiliated Hospital of Zhejiang University (Zhejiang, China), The Second Xiangya Hospital of Central South University (Hunan, China), The First Affiliated Hospital of Chongqing Medical University (Chongqing, China) |
| Study population | Screening population: 248  Number of patients to be included in the study: 210 (105 per study arm).  The actual number of patients included: 210 (108 in the perospirone group and 102 in the placebo group)  Number drop outs: 43 (23 in the perospirone group and 20 in the placebo group)  Number of patients to be evaluated: 177 in mITT (FAS) evaluation (87 in the perospirone group and 90 in the placebo group), and 167 with complete follow-up (85 in the perospirone group and 82 in the placebo group) |
| Inclusion criteria | - Meeting Diagnostic and Statistical Manual of Mental Disorders, Fifth Edition (DSM-5) criteria for MDD, confirmed by the Mini International Neuropsychiatric Interview (MINI); - The current episode duration of MDD must be ≤1 year; - Failed to respond adequately to at least one kind of antidepressant at therapeutic doses for at least 4 weeks, as indicated by a current score of at least 20 on the Montgomery-Asberg Depression Rating Scale (MADRS); - Aged 18 ~ 60 years; - Education >= 6 years and able to complete the cognitive tests; - Having the ability to understand and sign a written informed consent form prior to participation in any screening procedures. |
| Exclusion criteria | - Any other DSM-5 psychiatric disorder other than generalized anxiety disorder and social anxiety disorder diagnosed at present or lifetime; - Subjects with serious suicide ideation or attempts; - Subjects who have received treatments with adjunctive antidepressants and/or antipsychotic medications in the current depressive episode (small doses of benzodiazepines were permitted); - Subjects who have received non-drug treatments in past 6 months for more than 10 times, such as electroconvulsive therapy (ECT), repetitive transcranial magnetic stimulation (rTMS), and systemic psychotherapy; - DSM-5 alcohol or drug dependence; - History of head injury or loss of consciousness for more than 5 minutes; - Subjects with a history or current diagnose of major physical illness (such as thyroid disease, lupus erythematosus, diabetes, lung, liver and kidney damage, infection, major trauma and etc.); - Pregnant or breast-feeding; - Epilepsy, history of seizures or family history of epilepsy; - Receiving hormone therapy at present; - Transaminases (ALT or AST) 2 times or more above the upper limit of normal range; - QTc >= 430 msec (for males) or >= 450 msec (for females) in ECG; - Any subject with some other conditions that the researchers believe may affect the study results. |
| Study visits | Visits will be conducted at 0 weeks ± 2 days, 2 weeks ± 2 days, 4 weeks ± 2 days, and 8 weeks ± 2 days after enrollment in the trial. |
| Efficacy end points | Primary efficacy end points:   - MADRS response rate (defined as an improvement of ≥50% in the MADRS score from baseline) at week 4 and week 8. - MADRS remission rate (defined as a MADRS score of ≤10) at week 4 and week 8.   Secondary efficacy end points:   - Reduction rates in MADRS and Hamilton Anxiety Scale (HAMA) scores at each visit. - Change rates in each Clinical Global Impression (CGI) and Beck Suicidal Ideation Scale (BSI-CV) subscales scores at each visit. - Reduction rates in Pittsburgh Sleep Quality Index (PSQI), 16-item Quick Inventory of Depressive Symtomatology-Self-Report (QIDS-SR16), Generalized Anxiety Disorder 7-item Scale (GAD-7), and Snaith-Hamilton Pleasure Scale (SHAPS) scores at each visit. - Change rates in Sheehan Disability Scale (SDS) and Quality of Life Enjoyment Questionnaire - Short Form (Q-LES-Q-SF) scores at each visit. - Change rates in neurocognitive function test performance before and after treatment. |
| Safety end points | - Adverse events rated by Treatment-Emergent Symptom Scale (TESS) - Incidence of adverse events |

**1.2 Flowchart**


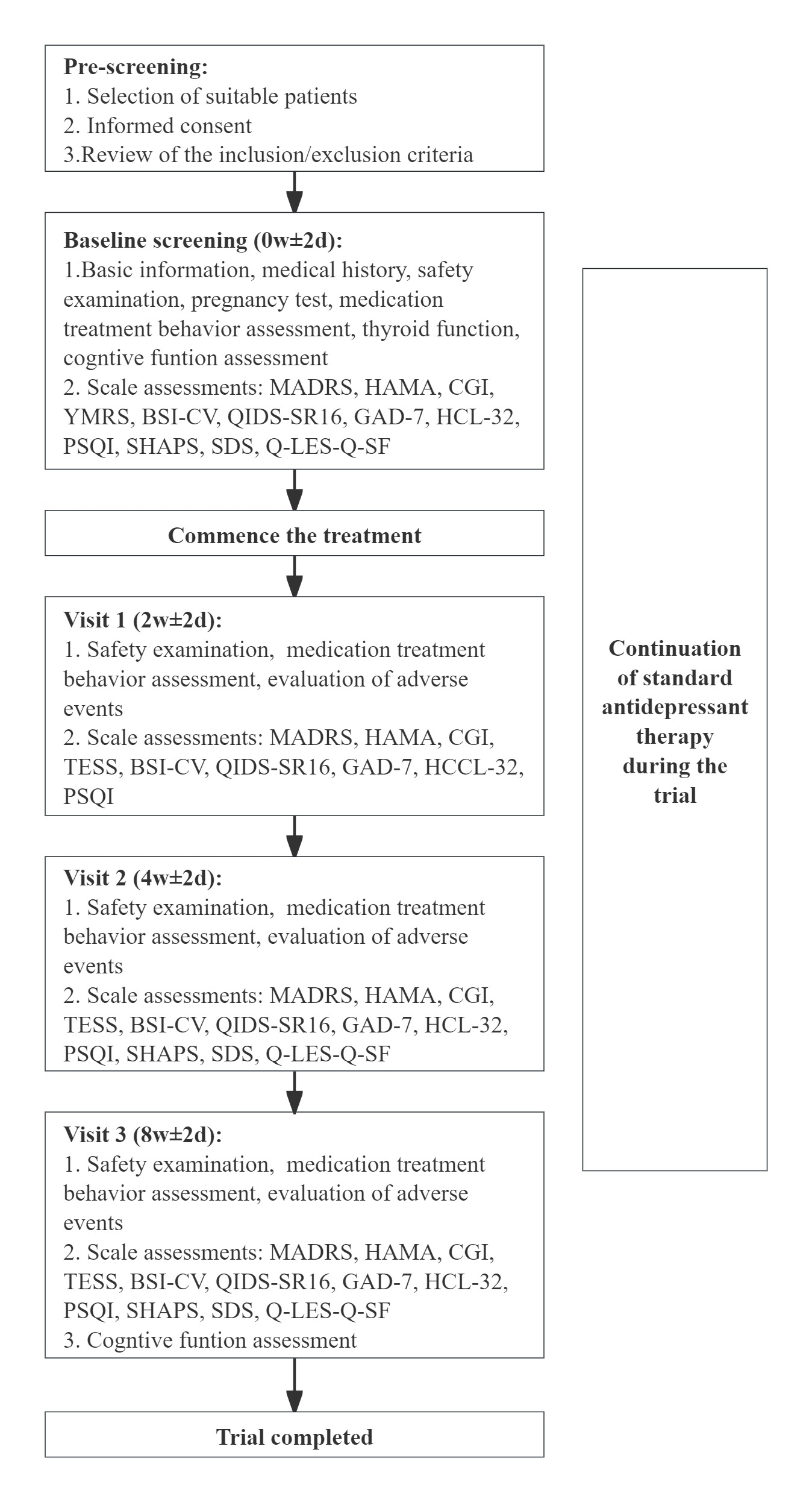


**Note:**

- Medical History includes current illness, past medical history, personal history, marital and reproductive history, menstrual history, and family history.
- Safety Assessments include vital signs, physical examination, complete blood count, blood glucose, blood lipids, liver and kidney function tests, urinalysis, and ECG.
- Specific examination items are detailed in the study schedule.
- Adverse events must be promptly recorded and assessed for their relationship with the treatment using perospirone tablets. Those assessed as definitely related, possibly related, or probably related are considered drug-related adverse reactions.

**1.3 Study schedule**

|  | Baseline screening (0w±2d) | Visit 1  (2w±2d) | Visit 2  (4w±2d) | Visit 3  (8w±2d) |
| --- | --- | --- | --- | --- |
| Inclusion/exclusion criteria | **×** |  |  |  |
| Informed consent | **×** |  |  |  |
| General information and medical history questionnaire^a^ | **×** |  |  |  |
| Vital signs^b^ | **×** | **×** | **×** | **×** |
| Physical examination | **×** | **×** | **×** | **×** |
| Medication treatment behavior | **×** | **×** | **×** | **×** |
| MADRS、HAMA、CGI | **×** | **×** | **×** | **×** |
| TESS |  | **×** | **×** | **×** |
| YMSR | **×** |  |  |  |
| BSI-CV | **×** | **×** | **×** | **×** |
| QIDS-SR16、GAD-7、HCL-32、PSQI | **×** | **×** | **×** | **×** |
| SHAPS、SDS、Q-LES-Q-SF | **×** |  | **×** | **×** |
| Cognitive function assessment^k^ | **×** |  |  | **×** |
| Complete blood count^c^、liver and kidney function^d^ | **×** | **×** | **×** | **×** |
| Blood glucose,lipid profile^e^ | **×** | **×** | **×** | **×** |
| Urinalysis^f^ | **×** | **×** | **×** | **×** |
| Electrocardiogram^h^ | **×** | **×** | **×** | **×** |
| Thyroid function^i^ | **×** |  |  |  |
| Pregnancy test (if applicable)^g^ | **×** |  |  |  |
| AE/SAE^j^ |  | **×** | **×** | **×** |
| Assessment of adverse event relatedness |  | **×** | **×** | **×** |
| a. General information includes name, age, gender, ethnicity, place of birth, height, weight, marital status, educational level, years of education, occupation, family economic status, household registration type during childhood, living arrangement, medication management, medical history provider, and health insurance status. Medical history includes current illness, past medical history, personal history, marital and reproductive history, menstrual history, and family history.  b. Vital signs include body temperature, pulse, respiration rate, and blood pressure.  c. Complete blood count (CBC) includes red blood cells (RBC), hemoglobin (Hb), white blood cells (WBC), absolute neutrophil count (ANC), lymphocytes (LYM), and platelets (PLT).  d. Liver and kidney function tests include alanine aminotransferase (ALT), aspartate aminotransferase (AST), alkaline phosphatase (ALP), gamma-glutamyl transferase (GGT), total protein (TP), total bilirubin (TBIL), direct bilirubin (DBIL), indirect bilirubin (IBIL), albumin (ALB), lactate dehydrogenase (LDH-L), creatinine (CRE), and blood urea nitrogen (BUN).  e. Lipid profile includes total cholesterol (TC), triglycerides (TG), high-density lipoprotein cholesterol (HDL-C), and low-density lipoprotein cholesterol (LDL-C).  f. Urinalysis includes pH, specific gravity (SG), glucose (GLU), protein (PRO), ketones (KET), blood (BLD), bilirubin (BIL), urobilinogen (URO), nitrite (NIT), and white blood cells (WBC).  g. Pregnancy test includes human chorionic gonadotropin (HCG) (serum or urine sample).  h. Electrocardiogram (ECG) status, including heart rate and QTc interval, should be recorded at each visit.  i. Thyroid function tests include free triiodothyronine (FT3), free thyroxine (FT4), and thyroid-stimulating hormone (TSH).  j. Adverse events (AEs) need to be recorded in detail and evaluated promptly. If any AEs occur during the treatment period, physical examination, blood sugar, blood lipids, CBC, liver and kidney function, urinalysis, and ECG should be rechecked according to the situation, and AEs should be evaluated and managed promptly, and reported to the research organization in a timely manner.  k. Cognitive assessment includes the Self-Rating Questionnaire for Cognitive Dysfunction in Depression (PDQ-D), Digit Span Test, Stroop Color-Word Interference Test, Trail Making Test, Hopkins Verbal Learning Test, and Digit-Symbol Substitution Test. | | | | |

1. Introduction
   1. **Background**

Major depressive disorder (MDD) has emerged as a prevalent condition that poses significant threats to both physical and mental health. In 2008, the World Health Organization (WHO) ranked MDD as the third leading cause of global disease burden and projected it to become the primary cause by 2030 [1]. According to epidemiological data, the prevalence of MDD in China is estimated between 3.5% and 5%, affecting approximately 54 million individuals nationwide; however, only 10% of these patients have received appropriate treatment [2]. The high incidence, low treatment-seeking rate, and high recurrence rate of MDD present substantial challenges for achieving long-term effective pharmacological management. While standard antidepressants are widely used in clinical settings, a considerable proportion of patients exhibit inadequate responses to at least one antidepressant. Emerging evidence from evidence-based medicine suggests that augmenting standard antidepressants with novel antipsychotic agents may offer an effective strategy for treating refractory depression.

Perospirone is a novel atypical antipsychotic originally developed in Japan and introduced in 2001. Perospirone demonstrates efficacy in addressing both positive and negative symptoms [3], primarily through activation of 5-HT1A receptors and antagonism of D2 and 5-HT2A receptors [4]. It also exhibits affinity for M receptors, α2 receptors, opioid receptors, and GABA receptors [5]. Preclinical studies indicate that 5-HT2A receptor antagonists enhance serotonin activity in the prefrontal cortex, playing a crucial role in alleviating depressive symptoms. Ziprasidone, a partial agonist of the 5-HT1A receptor, shares similar pharmacological properties with perospirone [6] and has demonstrated therapeutic efficacy in treatment-resistant major depression, with response rates of 32% and remission rates of 21% [7]. Perospirone's activation of 5-HT1A receptors increases dopamine release in the prefrontal cortex, thereby improving cognitive function [8, 9]. Research shows that perospirone has a higher affinity for 5-HT1A receptors compared to other antipsychotics [10], leading to enhanced non-verbal memory [11], general memory [12], and social cognitive functions [13]. Due to its antagonistic effect on 5-HT2A receptors, perospirone minimizes extrapyramidal side effects [14] and significantly improves sleep quality [15]. Additionally, perospirone does not affect serum prolactin levels [16], contributing to better patient compliance.

Perospirone were introduced to the Chinese market in 2008 and were included in the National Medical Insurance Catalogue in 2019. They are now commonly prescribed as atypical antipsychotics in clinical practice. This study, led by the Second Xiangya Hospital of Central South University, aims to evaluate the efficacy and safety of perospirone as an adjunctive therapy to antidepressants for patients with MDD who are resistant to conventional antidepressants, providing guidance for the safe and effective use of antidepressants in clinical settings.

- 1. **Risk/Benefit Assessment**

### 2.2.1 Identified Potential Risks

According to foreign research data, in the studies conducted prior to the approval of perospirone for marketing in Japan, 267 out of 429 cases (62.2%) experienced adverse events (AEs). The main AEs included extrapyramidal symptoms such as akathisia in 109 cases (25.4%), tremor in 65 cases (15.2%), rigidity in 52 cases (12.1%), and dysarthria in 45 cases (10.5%), as well as neuropsychiatric symptoms like insomnia in 93 cases (21.7%) and somnolence in 59 cases (13.8%). Abnormalities in laboratory tests included elevated prolactin levels in 27.5% of cases (28 out of 102), elevated CK (CPK) in 7.2% (23 out of 318), elevated AST (GOT) in 3.4% (13 out of 381), and elevated ALT (GPT) in 3.4% (13 out of 381).

Serious adverse events (SAEs) included malignant syndrome (incidence less than 0.1% to 5%), tardive dyskinesia (incidence less than 0.1 to 5%), paralytic intestinal obstruction (incidence unknown), syndrome of inappropriate antidiuretic hormone secretion (SIADH) (incidence less than 0.1 to 5%), convulsions (incidence unknown), and rhabdomyolysis (incidence unknown).

### 2.2.2 Identified Potential Benefits

Perospirone is an atypical antipsychotic that has been approved for market launch and has demonstrated favorable efficacy and safety profiles. Preliminary studies have indicated that the combination of atypical antipsychotics and antidepressants can alleviate depressive symptoms in patients with MDD who show inadequate response to standard antidepressants therapy, further improving treatment outcomes. Consequently, perospirone potentially enhance the effficacy of antidepressants to improve depressive symptoms in patients with MDD, improve social cognitive functions, and quality of life. Besides, it has a low incidence of extrapyramidal-related AEs and can significantly improve the sleep quality of patients.

The results of this study will guide clinical medication use, leading to more effective treatment for MDDpatients, who will potentially benefit from these findings.

### 2.2.3 Potential Risk/Benefit Assessment

Firstly, the risk of drug-related AEs for participants treating with perospirone in this study is minimal. Through rigorous monitoring by researchers and comprehensive training on relevant knowledge, any AEs are likely to be promptly identified and managed, thereby maintaining a significantly low incidence of both drug-related and non-drug-related AEs.

Secondly, participants will receive enhanced care during the study period and may benefit from cost reductions and subsidies, further mitigating potential risks. Existing literature indicates that atypical antipsychotics, when used adjunctively with antidepressants, can enhance the treatment efficacy for patients with MDD. Thus, perospirone, as an atypical antipsychotic, has demonstrated positive effects in treating MDD, offering therapeutic benefits to participants.

Furthermore, there is limited evidence on the use of perospirone as an augmentation strategy for patients with MDD who are resistant to at least one antidepressant for at least 4 week. This study aims to establish the efficacy and safety of perospirone in treating MDD through standardized and rigorous clinical trial, thereby generating evidence-based data to guide clinical practice and optimize treatment protocols for MDD patients who are resistant to standard antidepressant therapy.

Consequently, the anticipated benefits of this trial are expected to outweigh the potential risks.

1. Research Objectives and Endpoints

Perospirone Hydrochloride Tablets were launched in Japan in 2001 and in China in 2008, and were included in China's National Medical Insurance Catalogue in 2019. Given the particularity of long-term medication use for patients with MDD, the efficacy and safety of the drug are especially important. To better serve clinical practice, we plan to conduct a study titled "Efficacy and safety of augmentation pharmacotherapy with perospirone for major depressive disorder patients resistant to antidepressants: a randomized, double-blind, placebo-controlled trial"

The objectives, endpoints and the explanations of the research are shown in the table below:

| Objective | Endpoint | Endpoint explanation |
| --- | --- | --- |
| Primary efficacy end points | | |
| Response rate | MADRS remission rate (defined as a MADRS score of ≤10) at week 4 and week 8. | To evaluate the clinical efficacy of perospirone as an adjunctive antidepressant treatment for MDD by assessing the proportion of patients with ≥50% reduction in MADRS scores at Week 4 and Week 8. |
| Remission rate | MADRS remission rate (defined as a MADRS score of ≤10) at week 4 and week 8. | To evaluate the clinical efficacy of perospirone as an adjunctive antidepressant treatment for depression by assessing the proportion of patients with MADRS scores ≤10 at Week 4 and Week 8. |
| Secondary efficacy end points | | |
| Clinical symptoms improvement | Reduction rates in MADRS and QIDS-SR16 scores at each visit. | To evaluate the efficacy of perospirone as an adjunctive antidepressant treatment for depression in improving depressive symptoms by comparing the reduction rates of MADRS and QIDS-SR16 scores at each follow-up visit. |
|  | Reduction rates in HAMA and GAD-7 scores at each visit. | To evaluate the efficacy of perospirone as an adjunctive antidepressant treatment for depression in improving anxiety symptoms by comparing the reduction rates of HAMA and GAD-7 scores at each follow-up visit. |
|  | Reduction rates in PSQI scores at each visit. | To evaluate the efficacy of perospirone as an adjunctive antidepressant treatment for depression in improving sleep quality by assessing the reduction rates of PSQI at each follow-up visit. |
|  | Reduction rates in SHAPS scores at each visit. | To evaluate the efficacy of perospirone as an adjunctive antidepressant treatment for depression in improving the symptoms of anhedonia by comparing the change rates of SHAPS scores at each follow-up visit. |
|  | Reduction rates in CGI and BSI  -CV scores at each visit. | To evaluate the efficacy of perospirone as an adjunctive antidepressant treatment for depression in alleviating symptoms of suicidal ideation and improving overall clinical impression by comparing the change rates of CGI and BSI-CV subscale scores at each follow-up visit. |
| Quality of life and social functionings | Change rates in SDS and Q-LES-Q-SF scores at each visit | To evaluate the efficacy of perospirone as an adjunctive antidepressant treatment for depression in improving patients’ quality of life and social functionings by comparing the change rates of SDS and Q-LES-Q-SF scores at each follow-up visit. |
| Cognitive performance | Change rates in neurocognitive test scores (including Digit Span Test, Stroop Color Word Test, Trail Making Test, Hopkins Verbal Learning Test, and Digit-Symbol Substitution Test) and Self-Rating Questionnaire for Cognitive Dysfunction-Depression (PDQ-D) Score before and after treatment. | To evaluate the efficacy of perospirone as an adjunctive antidepressant treatment for depression in improving patients’ cognitive performance by comparing the change rates of each neurocognitive test scores at each follow-up visit. |
| Safety endpoints | | |
| Safety | TESS scores | To evaluate the safety of perospirone as an adjunctive antidepressant treatment for patients with MDD when used in combination with antidepressants, utilizing the TESS to assess the severity of AEs and the measures taken for their management. |
|  | Incidence of AEs | To evaluate the safety of perospirone as an adjunctive antidepressant treatment for patients with MDD when used in combination with antidepressants, based on the incidence of AEs during treatment. This assessment includes the overall incidence of AEs, the incidence of extrapyramidal symptoms (EPS), liver and kidney function, and ECG abnormalities. |

1. Study Design

## Overall design

This study is a randomized, double-blind, placebo-controlled, parallel-group trial. Participants that meet all the inclusion criteria and do not meet any exclusion criteria will be enrolled in the study based on the their willingness. After enrollment, baseline data of the participants will be recorded, and medication treatment will commence. Detailed records of the treatment and any concomitant medications will be maintained, and participants will be visited and assessed at the specified visit time points.

This study is led by Xiangya Second Hospital of Central South University in collaboration with six centers. A total of 210 participants are expected to be enrolled, with 105 in the treatment group and 105 in the control group. The study aims to investigate the efficacy and safety of perospirone as an adjunctive treatment for antidepressants in the treatment of depression.

## The scientific rationale of the study design

The study employs a variety of widely used clinical scales related to depression to ensure the sensitivity of the research results. Different scales for evaluating depressive symptoms are selected to assess the improvement of various depressive symptoms (e.g., core depressive symptoms, sleep disturbance, cognitive performance impairmment) by the treatment medications. Additionally, different patients with MDD may have different responses to the treatment drugs and varying tolerances to AEs. Therefore, physicians will choose appropriate treatment methods for participants based on specific clinical situations, and detailed records of dosage adjustments and concomitant medications will be maintained.

## The rationale for drug dosage selection

According to the instructions from December 29, 2015, perospirone should be taken orally after meals. The initial dose for adults is 4 mg per administration, three times daily, with the dose gradually increased according to the patient's response and tolerance. The maintenance dose ranges from 12 mg to 48 mg per day, divided into three doses after meals. The dosage should be adjusted appropriately based on age and symptoms, and the maximum daily dose should not exceed 48 mg.

## The definition of study termination

In this study, a participant is considered to have completed the study if they have finished all scheduled visits and no further examinations are required. Alternatively, if a participant is confirmed as lost to follow-up, they are also considered to have completed the study.

The study is defined as completed when all participants across all centers have finished their respective study procedures, the data have been fully analyzed, and the study report has been generated.

## Randomization method

Randomization will be conducted using a random number table, with fixed enrollment across multiple centers. It is expected that each of the 7 centers will enroll 30 patients with depression (excluding potential dropouts). Considering potential dropouts, each center will be provided with 36 random numbers. The randomization codes (blinding codes) will be distributed together with the study medications and will be kept solely by the drug dispenser (who will not participate in any assessments). Other personnel, including clinical physicians, assessors, clinical research assistants, and others, will remain unaware of the randomization codes and will be unable to distinguish which medication the participants will receive.

## Blinding, unblinding and breaking the blind operation procedures

### 4.6.1 Blinding code storage

The blinding of the investigational medication will be conducted by specialized personnel who are not directly involved in the clinical trial. These personnel will assign numbers to the investigational medication according to the master randomization table. For each randomization number, a blinding code letter will be prepared, containing information on the participant's group assignment and medication details. The blinding codes will be kept by the principal investigator and a designated person from the sponsor's organization, and they must not be opened until the end or termination of the study.

### 4.6.2 Blinding code storage

During the study period, if no emergency occurs for the participants, the unblinding will proceed according to the standard procedure:

After the completion of the trial, following verification of the Case Report Form (CRF) and signatures, a Level 1 unblinding will be conducted to determine the group assignments of the participants. This step is essential for performing the statistical analysis.

Following the completion of the statistical analysis and the generation of the statistical conclusions, a Level 2 unblinding will be performed to identify the treatment and control groups. This step will allow for the evaluation of the efficacy of the investigational medication.

### 4.6.3 Unblinding under emergency

In the event of an urgent medical incident where the investigator and the principal investigator determine that the participant cannot receive adequate treatment, the following emergency unblinding procedures will be implemented:

1. Notify the sponsor: prior to unblinding, the sponsor will be informed.
2. Unblind based on emergency letter: the treatment assignment for the participant will be revealed based on the information provided in the emergency letter.
3. Complete unblinding documentation: the investigator will complete the unblinding record form and document the unblinding in the CRF.
4. Post-unblinding notification: if the sponsor cannot be contacted prior to unblinding, the investigator will notify the sponsor immediately after unblinding.

The emergency letters will be collected along with the CRF at the end of the trial for blind review.

1. Study Population

## Inclusion criteria

1. Meeting Diagnostic and Statistical Manual of Mental Disorders, Fifth Edition (DSM-5) criteria for MDD, confirmed by the Mini International Neuropsychiatric Interview (MINI);

2. The current episode duration of MDD must be ≤1 year;

3. Failed to respond adequately to at least one kind of antidepressant at therapeutic doses for at least 4 weeks, as indicated by a current score of at least 20 on the Montgomery-Asberg Depression Rating Scale (MADRS);

4. Aged 18 ~ 60 years;

5. Education >= 6 years and able to complete the cognitive tests;

6. Having the ability to understand and sign a written informed consent form prior to participation in any screening procedures.

## Exclusion criteria

1. Any other DSM-5 psychiatric disorder other than generalized anxiety disorder and social anxiety disorder diagnosed at present or lifetime;

2. Subjects with serious suicide ideation or attempts;

3. Subjects who have received treatments with adjunctive antidepressants and/or antipsychotic medications in the current depressive episode (small doses of benzodiazepines were permitted);

4. Subjects who have received non-drug treatments in past 6 months for more than 10 times, such as electroconvulsive therapy (ECT), repetitive transcranial magnetic stimulation (rTMS), and systemic psychotherapy;

5. DSM-5 alcohol or drug dependence;

6. History of head injury or loss of consciousness for more than 5 minutes;

7. Subjects with a history or current diagnose of major physical illness (such as thyroid disease, lupus erythematosus, diabetes, lung, liver and kidney damage, infection, major trauma and etc.);

8. Pregnant or breast-feeding;

9. Epilepsy, history of seizures or family history of epilepsy;

10. Receiving hormone therapy at present;

11. Transaminases (ALT or AST) 2 times or more above the upper limit of normal range;

12. QTc >= 430 msec (for males) or >= 450 msec (for females) in ECG;

1. Any subject with some other conditions that the researchers believe may affect the study results.

## Lifestyle precautions

During the study period, the use of caffeine, alcohol, or tobacco is prohibited. Participants are required to fast for 8 hours before blood sampling for laboratory tests. Strenuous exercise is not allowed during the trial. However, participants may engage in some light and enjoyable activities, such as watching TV, reading, or taking a walk.

## Screening failure

Screening failure is defined as a participant who consents to participate in the clinical trial and signs the informed consent form, but during the screening process, one or more inclusion criterias are not met, or one or more exclusion criterias are met. Participants with screening failure will not be accepted for study interventions or enrolled in the study. However, information of participants with screening failure, such as demographic data, will be retained, and their screening numbers will also be preserved.

## Recruitment and retention strategies

This study will recruit participants nationwide. All investigators involved in the study across centers will undergo systematic training to become familiar with the inclusion and exclusion criteria, and to rapidly identify potential participants who are likely to meet the study requirements. Selected participants will receive financial compensation and a reduction in some examination costs. The importance of study visits will be thoroughly explained, and visit compensation or rewards will be established.

1. Study Intervention

## Management of study intervention

### 6.1.1 Description of study intervention

Study Group (Perospirone Group): Perospirone + SSRIs/SNRIs

Control Group: Placebo + SSRIs/SNRIs

The placebo is indistinguishable from perospirone in terms of appearance, shape, odor, and specifications.

### 6.1.2 Dosing and administration

Study Group (Perospirone Group): Oral administration of perospirone, with a recommended dose of 8–24 mg/day, adjusted according to patients’ responses and tolerance, within a dose range of 4–48 mg/day; SSRIs/SNRIs will be maintained at the original therapeutic dose, with a treatment duration of 8 weeks.

Control Group: Oral administration of placebo, matched in dose to perospirone; SSRIs/SNRIs will be maintained at the original therapeutic dose, with a treatment duration of 8 weeks.

## Management of investigational medications

### 6.2.1 Supply and dispensing of investigational medications

The investigational medications (including perospirone and placebo) will be provided free of charge by Livzon (Group) Pharmaceutical Factory. During the transportation of the investigational medications to the institution or department, the transportation process, temperature, and duration should be inspected and recorded, with the records being kept by the receiving party.

For outpatients, investigational medications will be dispensed based on a prescription. The pharmacy administrator or medical staff will instruct participants on the method of administration and other precautions, and advise them to bring all remaining investigational medications and empty packaging to their next visit. For inpatients, investigational medications will be dispensed based on a medical order or prescription, and medical staff will strictly adhere to the study protocol when administering the products.

At the end of the study, all remaining investigational medications and empty packaging must be collected and returned.

### 6.2.2 Formulation, appearance, packaging, and labeling of investigational medications

Perospirone and placebo are both manufactured by Livzon Pharmaceutical Factory of Livzon Group. They are white tablets, packaged in aluminum-plastic blister packs, and labeled with the indication "For Clinical Research Use Only." Both products are identical in appearance, odor, specifications, and packaging. As this study is double-blind, the medications are also marked with special labels that allow identification of perospirone and placebo through emergency letters in case of urgent situations.

### 6.2.3 Storage conditions

Protect from light, store in a sealed container, and keep at room temperature.

## Methods to reduce deviations

All investigators across participating centers will undergo standardized training to strictly adhere to the inclusion and exclusion criteria for participant selection and to standardize evaluation criteria, thereby minimizing potential deviations.

## Patient compliance with study interventions

Investigators should ensure the comprehensive preservation of participants' original medication records and medical files, as these documents serve as crucial evidence for assessing participant compliance.

## Concomitant medications

### 6.5.1 Permitted concomitant medications

This study will not interfere with the clinical physicians' choice of medications. All conventional treatment methods and medications for complications that are not explicitly prohibited are permitted. During the screening phase, if a participant is required to continue taking medication or receiving other treatments for a comorbid condition, such treatment may continue. Unless the comorbid condition worsens or improves, the dosage should not be altered during the trial. However, the medication name (or other therapy name), dosage, reason for use, frequency, and timing must be recorded in the CRF for analysis and reporting during the study summary. No new psychotherapy or cognitive-behavioral therapy should be initiated during the study period. For participants with severe sleep disorders, the concurrent use of low-dose benzodiazepines may be permitted.

### 6.5.2 Prohibited and cautionary concomitant medications

1. Contraindicated for use with adrenaline.

2. Use with caution when co-administered with central nervous system depressants (e.g., barbiturates), as there is a potential for additive central nervous system depression. Dose adjustment may be necessary.

3. Use with caution when co-administered with dopaminergic agents (e.g., levodopa, bromocriptine mesylate), as there is a potential for antagonistic effects. Dose adjustment may be necessary.

4. Use with caution when co-administered with antihypertensive agents, as there is a potential for additive antihypertensive effects. Dose adjustment may be necessary.

5. Co-administration with domperidone or metoclopramide may lead to endocrine dysfunction or extrapyramidal symptoms.

6. Use with caution when co-administered with H2 receptor antagonists (e.g., cimetidine), as there is a potential for additive inhibition of gastric acid secretion. Close monitoring and cautious dosing are recommended.

7. Use with caution when co-administered with selective inhibitors of CYP3A4 (e.g., macrolide antibiotics), as this may increase plasma concentrations of this medication, potentially leading to an increased incidence of adverse reactions. Close monitoring and cautious dosing are recommended.

8. Use with caution when co-administered with drugs metabolized by CYP3A4 (e.g., cisapride, triazolam), as this may increase the incidence of adverse reactions for both medications. Close monitoring and cautious dosing are recommended.

9. Other medications that the investigator deems may affect the study outcomes.

### 6.5.3 Emergency drugs

The risk of requiring resuscitation is low in this study. However, each research center should still have conventional resuscitation medications on standby to address extremely rare occurrences of anaphylactic shock and other emergencies.

1. Termination of Study Intervention and Discontinuation/Withdrawal of Participants

## Termination of study intervention

If intolerable AEs occur in participants, or if AEs that the investigator deems may increase the risk to participants are observed, the use of investigational medication should be discontinued. The reasons and timing for the discontinuation of the intervention should be documented in detail.

Discontinuation of the study intervention does not mean discontinuation of the study itself. Participants should continue to follow the procedures outlined in the study protocol. If clinically significant changes in the laboratory tests occur after enrollment (including but not limited to deviations from baseline levels), the investigator or a qualified designated person will determine whether changes in participant management are necessary. Any new clinically relevant findings will be reported as AEs.

## Discontinuation/withdrawal of participants

Participants have the right to withdraw from the study at any time based on their own wishes. Investigators may request discontinuation or withdrawal of participants from the study for the following reasons:

1.Pregnancy.

2.Poor compliance with the study intervention.

3.Occurrence of a clinical AE, abnormal laboratory test results, or other medical conditions where continued participation in the study would not be in the best interest of the participant.

4.Disease progression that requires discontinuation of the intervention.

5.The participant meets exclusion criteria (newly identified or previously undetected) and is no longer eligible to continue in the study.

6.The participant is unable to receive continuous treatment with perospirone for 8 weeks.

The reasons and timing for discontinuation or withdrawal from the study should be documented in the CRF. Participants who have provided informed consent and been randomized but have not yet received the study intervention may be replaced. Participants who have provided informed consent, been randomized, and received the study intervention, and subsequently withdraw or are withdrawn/discontinued from the study, cannot be replaced.

## Lost to follow-up

During the post-treatment follow-up phase, if a participant does not return to the research center for the scheduled visit and the research center staff are unable to contact them, the participant will be considered lost to follow-up. Similarly, during the survival follow-up phase, if it is not possible to contact the participant or their family members to confirm their survival status and complete the survival follow-up, the participant will also be considered lost to follow-up.

If a participant does not return to the research center for the specified study visit, the following actions must be taken:

The research center will attempt to contact the participant and reschedule the missed visit as soon as possible within one week. The importance of adhering to the visit schedule will be explained to the participant, and it will be confirmed whether the participant is willing and/or should continue to participate in the study.

Before a participant is deemed lost to follow-up, the investigator or designated personnel will make every effort to re-establish contact with the participant (e.g., making three phone calls, sending a registered letter to the participant's latest mailing address if necessary, or using other locally effective means of contact). These attempts to contact the participant should be documented in the participant's medical record or study file.

If the participant still cannot be contacted, they will be considered lost to follow-up and withdrawn from the study.

1. Study Assessments and Procedures

## Efficacy assessments

### 8.1.1 Primary efficacy assessments

- **MADRS response rate**: the proportion of participants with a ≥50% reduction in the MADRS score from baseline at week 4 and week 8.
- **MADRS remission rate**: The proportion of participants with an MADRS score ≤10 at week 4 and week 8.

This study evaluates the efficacy of perospirone as an adjunctive antidepressant treatment for MDD by comparing the response rates and remission rates between the study group and the control group.

### 8.1.2 Secondary efficacy end points assessments

1. To evaluate the efficacy of perospirone as an adjunctive antidepressant treatment for depression in improving depressive symptoms by comparing the reduction rates of MADRS and QIDS-SR16 scores at each follow-up visit.

2. To evaluate the efficacy of perospirone as an adjunctive antidepressant treatment for depression in improving anxiety symptoms by comparing the reduction rates of HAMA and GAD-7 scores at each follow-up visit.

3. To evaluate the efficacy of perospirone as an adjunctive antidepressant treatment for depression in improving sleep quality by assessing the reduction rates of PSQI at each follow-up visit.

4. To evaluate the efficacy of perospirone as an adjunctive antidepressant treatment for depression in improving the symptoms of anhedonia by comparing the change rates of SHAPS scores at each follow-up visit.

5. To evaluate the efficacy of perospirone as an adjunctive antidepressant treatment for depression in alleviating symptoms of suicidal ideation and improving overall clinical impression by comparing the change rates of CGI and BSI-CV subscale scores at each follow-up visit.

6. To evaluate the efficacy of perospirone as an adjunctive antidepressant treatment for depression in improving patients’ quality of life and social functionings by comparing the change rates of SDS and Q-LES-Q-SF scores at each follow-up visit.

7. To evaluate the efficacy of perospirone as an adjunctive antidepressant treatment for depression in improving patients’ cognitive performance by comparing the change rates of each neurocognitive test scores at each follow-up visit.

## Safety assessments

The safety profile of perospirone as an adjunctive antidepressant treatment for MDD will be comprehensively evaluated using the TESS and the incidence of AEs.

The TESS include symptoms and signs of common AEs, as well as several laboratory test results. Each symptom will be assessed in terms of AE severity, the relationship between AEs and investigational medications, and the corresponding management measures.

The evaluation of AEs incidences will include both the overall incidence of AEs and specific incidences related to liver and kidney function, electrocardiogram (ECG) abnormalities, and other relevant aspects. This assessment will involve monitoring and recording all AEs and SAEs, routine blood and biochemical parameters, vital signs, physical examinations, and all treatment methods and concomitant medications. Safety indicators will encompass vital signs, physical examinations, clinical laboratory parameters, AEs, and reasons for early withdrawal. For vital signs and laboratory parameters, the proportion of abnormal test results will be compared between the study group and the control group.

The incidence of AEs will be expressed as the ratio of participants experiencing AEs during the trial to the total number of participants eligible for safety evaluation. AEs will be summarized according to treatment group, body system, and events within each system.

The assessment of AEs includes classification, grading, relationship to the medications, management measures, and outcomes.

## Trial procedure

### Baseline screening

At the baseline screening, the investigator is required to complete the collection of the patient's general information and medical history, vital signs, physical examination, and description of antidepressant treatment behaviors. Patients are required to undergo laboratory tests, including complete blood count (CBC), liver and kidney function tests, blood glucose, lipid profile, urinalysis, pregnancy test, electrocardiogram (ECG), and thyroid function tests. Patients are also required to cooperate in completing scale assessments, including the Montgomery-Åsberg Depression Rating Scale (MADRS), Hamilton Anxiety Rating Scale (HAMA), Clinical Global Impression (CGI), Young Mania Rating Scale (YMRS), Chinese version of the Beck Suicide Ideation Scale (BSI-CV), Pittsburgh Sleep Quality Index (PSQI), Quick Inventory of Depressive Symptomatology – Self-Report (QIDS-SR16), Generalized Anxiety Disorder scale (GAD-7), Chinese version of the Hypomania Checklist (HCL-32), Snaith-Hamilton Pleasure Scale (SHAPS), Sheehan Disability Scale (SDS), Quality of Life Enjoyment and Satisfaction Questionnaire – Short Form (Q-LES-Q-SF), Perceived Deficits Questionnaire – Depression (PDQ-D), and neurocognitive tests (including Digit Span Test, Stroop Color-Word Interference Test, Trail Making Test, Hopkins Verbal Learning Test, and Digit-Symbol Substitution Test).

### Visit 1

Completion the assessments of vital signs, physical examination, and description of antidepressant treatment behaviors; completion of laboratory tests, including CBC, liver and kidney function tests, blood glucose, lipid profile, urinalysis, and ECG; completion of scale assessments, including the MADRS, HAMA, CGI, BSI-CV, PSQI, QIDS-SR16, GAD-7, HCL-32, and the TESS.

### Visit 2

Completion the assessments of vital signs, physical examination, and description of antidepressant treatment behaviors; completion of laboratory tests, including CBC, liver and kidney function tests, blood glucose, lipid profile, urinalysis, and ECG; completion of scale assessments, including the MADRS, HAMA, CGI, TESS, BSI-CV, PSQI, QIDS-SR16, GAD-7, HCL-32, SHAPS, SDS and Q-LES-Q-SF

### Visit 3

Completion the assessments of vital signs, physical examination, and description of antidepressant treatment behaviors; completion of laboratory tests, including CBC, liver and kidney function tests, blood glucose, lipid profile, urinalysis, and ECG; completion of scale assessments, including the MADRS, HAMA, CGI, TESS, BSI-CV, PSQI, QIDS-SR16, GAD-7, HCL-32, SHAPS, SDS and Q-LES-Q-SF; completion of neurocognitive tests, including Digit Span Test, Stroop Color-Word Interference Test, Trail Making Test, Hopkins Verbal Learning Test, and Digit-Symbol Substitution Test.

## Adverse event (AE) and serious adverse event (SAE)

### Definition of adverse event (AE)

An Adverse event (AE) is defined as any untoward medical occurrence in a participant associated with the use of the study medication or occurring during the study, regardless of whether there is a causal relationship with the study medication or treatment process.

Endpoint events are not considered AEs unless, based on the investigator's best clinical judgment, considering the course, severity, or other characteristics of the event, it should be regarded as an exception in this clinical context.

Any AE, regardless of its severity or whether it is related to the study medication, should be recorded in the CRF from the time the participant signs the informed consent form until the end of the last scheduled visit as planned in the study protocol. The investigator should specifically describe the timing of the event, its severity, any actions taken regarding the study medication, symptomatic treatment provided, outcomes, and his/her judgment on whether the AE might have been caused by the study medication.

Laboratory test results, vital signs, or ECG abnormalities will be recorded as AEs if they are medically related to the study medication, such as when accompanied by clinical symptoms, necessitating symptomatic treatment, leading to discontinuation of the medication, or resulting in serious consequences.

### Definition of serious adverse event (SAE)

An adverse event will be considered a Serious Adverse Event (SAE) if it meets any of the following criteria:

1. Results in death.
2. Is life-threatening (defined as an event that poses an immediate threat to the patient's life at the time of occurrence, rather than a hypothetical situation where the event would lead to death if it were more severe).
3. Results in persistent or significant disability/incapacity.
4. Requires inpatient hospitalization or prolongation of existing hospitalization.
5. Involves congenital anomaly/birth defect.
6. Other important medical events.

### Severity of AEs

The severity of Adverse Events (AEs) is evaluated according to the general grading principles of the National Cancer Institute's Common Terminology Criteria for Adverse Events (CTCAE v5.0), which are divided into five levels:

**Grade 1**: Mild; asymptomatic or only mildly symptomatic; detected only clinically or by diagnostic studies; intervention not indicated.

**Grade 2**: Moderate; minimal, focal, or non-invasive intervention indicated; limited age-related instrumental activities of daily living.

**Grade 3**: Severe or medically significant but not immediately life-threatening; inpatient hospitalization or prolongation of existing hospitalization indicated; disability; self-care activities of daily living limited.

**Grade 4:** Life-threatening; urgent intervention indicated.

**Grade 5**: Death related to the AE.

### Management of AEs

When an AE occurs, the investigator will take appropriate management measures based on the severity of the event. If the symptoms are mild and consistent with the common toxic side effects previously observed with perospirone, dose reduction, temporary discontinuation of the drug, and close monitoring may be implemented, with timely adjustments as needed. If the symptoms are severe or beyond the scope of common toxic side effects, the drug should be discontinued immediately. Additional necessary laboratory tests and assessments should be conducted to determine the severity of the AE and its corresponding impact. Symptomatic treatment will be provided based on clinical manifestations and test results. The costs associated with these additional tests and assessments will be covered by the sponsor. Regardless of the severity of the adverse event, the investigator must document the entire process in detail.

If the occurrence of an AE compromises the rights and safety of the participant, the sponsor will assume relevant responsibilities in accordance with national laws and regulations, covering the costs of related treatments and providing appropriate financial compensation. In the event of rare or new SAEs that fall outside the expected scope, the sponsor will organize consultations with the patient and their family members to provide reasonable financial compensation.

### Causality assessments

Physicians who examine and assess participants must evaluate the causality of all AEs in relation to the study intervention based on temporal relationships and their clinical judgment. The certainty of causality will be graded using the following categories:

**Definitely related to the study drug:** There is evidence of taking the investigational medication; the temporal sequence between the occurrence of the AE and the intake of the study drug is credible; the AE is more reasonably explained by the study drug than by other causes; positive dechallenge reaction; positive rechallenge reaction; the pattern of the AE is consistent with prior knowledge of this or similar drugs.

**Probably related to the study drug:** There is evidence of taking the study drug; the temporal sequence between the occurrence of the AE and the intake of the study drug is credible; the AE is more reasonably explained by the study drug than by other causes; positive dechallenge reaction.

**Possibly related to the study drug:** There is evidence of taking the study drug; the temporal sequence between the occurrence of the AE and the intake of the study drug is credible; the AE may be caused by the study drug but could also be due to other causes; positive dechallenge reaction.

**Possibly unrelated to the study drug:** There is evidence of taking the study drug; the AE is more likely due to other causes; negative or equivocal dechallenge reaction; negative or equivocal rechallenge test.

**Definitely unrelated to the study drug:** The participant did not take the study drug; or the temporal sequence between the occurrence of the AE and the intake of the study drug is not credible; or there are other significant causes that could explain the AE.

### Requirements for AE recording and follow-up

When participants receiving medical services during visits or monitors conducting reviews, the occurrence of AEs or SAEs should draw the attention of researchers.

All AEs that do not meet the criteria for SAEs will be recorded in the CRF, including both local and systemic reactions. Information to be collected includes a description of the event, onset time, assessment of severity by a physician, relationship to the investigational medication (which can only be assessed by trained personnel with diagnostic authority), and the time when the event is resolved or stabilized. All AEs occurring during the study must be comprehensively documented, regardless of their relationship to the study intervention. All AEs should be followed up until reasonably resolved.

Medical conditions at the time of participant screening should be considered baseline and not reported as AEs. However, if the participant's condition worsens at any time, it should be recorded as an AE.

Changes in the severity of AEs should be documented to assess the duration of the event at different severity levels. Intermittent AEs require recording of onset time and the duration of each onset.

From the time of signing the informed consent form to the end of the trial, investigators should record all AEs/SAEs. At each visit, researchers should inquire about AEs/SAEs that occurred since the last visit and follow up on the outcome of the events until they are resolved or stabilized.

### AE reporting

Investigators should inform participants to truthfully report any changes in their condition following medication use. Physicians should avoid leading questions. While observing treatment efficacy, attention should also be paid to AEs or unexpected toxic side effects (including symptoms, signs, and laboratory test results). Investigators should analyze the causes, make judgments, and calculate the incidence of AEs.

For AEs occurring during the study, symptoms, severity, onset time, duration, management measures, and course of the event should be documented in the CRF. The relationship between the AEs and the investigational medication should be evaluated, and the form should be signed by the investigator with the date noted.

### SAE reporting

In the event of a SAE occurring during the study, the investigator shall immediately (usually within 24 hours of becoming aware) provide the sponsor with a written report of all SAEs, followed by a timely, detailed, and written follow-up report. SAE reports and follow-up reports should include the participant's identification code used in the clinical trial, rather than the participant's real name, national identity number, address, or other personal identification information.

Upon receiving any safety-related information from any source, the sponsor shall immediately conduct a comprehensive analysis, assessment, and judgment of the SAE. If it meets the definition of a Suspected Unexpected Serious Adverse Reaction (SUSAR), the sponsor is required to send the processed SUSAR report and follow-up report to the investigator within the specified timeframe.

For reports involving fatal events, the investigator shall provide the sponsor and the ethics committee with any additional required information, such as autopsy reports and final medical reports.

### Reporting AEs/SAEs to participants

After the occurrence of an AE/SAE, active management is required. Following the investigator's assessment of causality, timely communication with the patient is necessary to seek the patient’s opinion on whether to continue participating in the study and receiving the intervention.

### Adverse event of special interest

Not applicable.

### Pregnancy reporting

Pregnancy is not considered an AE. However, if pregnancy is confirmed during the treatment period, the study intervention will be discontinued, but participants may continue to be followed up.

The timeline for reporting pregnancy events is the same as that for SAEs, and follow-up should continue until the pregnancy outcome (e.g., termination of pregnancy, childbirth). Investigators are required to report pregnancy events to the sponsor within the specified timeframe.

If subsequent events occur during pregnancy, they should be managed as SAEs: fetal/neonatal congenital abnormalities or deformities, spontaneous abortion, termination of pregnancy for medical reasons.

Both male and female participants should take effective contraceptive measures after enrolling in the study.

## Unanticipated problem (UP)

### Definition of unanticipated problem (UP)

The risks to participants or others arising from UP must be carefully evaluated. Generally, these include any event, process, or outcome that meets all of the following criteria:

1. **Unexpected:** The event is unexpected in terms of its nature, severity, or frequency, given: (a) the research procedures described in the protocol-related documents, such as the study protocol approved by the Institutional Review Board (IRB) and the informed consent form; and (b) the characteristics of the population being studied.
2. **Related or possibly related:** The event is related or possibly related to participation in the research ("Possibly related" means that there is a reasonable possibility that the event, process, or outcome was caused by procedures involved in the study).
3. **Greater risk of harm:** The event suggests that participants or others may be at greater risk of harm than was previously known or recognized (including physical, psychological, economic, or social harm).

### UPs reporting

Investigators will report UPs to the IRB reviewers and the Principal Investigator (PI). UP reports will include the following information:

1. **Protocol identification information:** Protocol name and number, PI name, and IRB project number.
2. **Detailed description of the UP:** A detailed description of the event, including any associated circumstances or outcomes.
3. **Basis for identifying the UP:** A description of how the event, associated circumstances, or outcomes were identified as a UP.
4. **Description of any protocol changes or corrective actions:** A description of any changes to the protocol or other corrective actions that have been taken or proposed in response to the UP.

To meet the need for prompt reporting, UPs will be reported according to the following schedule:

1.**UPs related to SAEs**: UPs related to SAEs will be reported to the IRB and the study sponsor within 24 hours of the investigator becoming aware of the event.

2.**Any Other UPs**: Any other UPs will be reported to the IRB and the study sponsor within 24 hours of the investigator becoming aware of the event.

3.**Reporting to regulatory authorities:** Within 24 hours of receiving a report of a problem from the investigator, the IRB should report all UPs to the provincial, autonomous region, or municipal drug administration authorities and the Drug Adverse Reaction Evaluation Center.

### Suspected unexpected serious adverse event (SUSAR) reporting

Suspected Unexpected Serious Adverse Reaction (SUSAR) refers to a serious adverse event that is suspected and unexpected, based on the nature and severity of the clinical presentation exceeding the information documented in the Investigator's Brochure for the investigational drug, the package insert for marketed drugs, or the Summary of Product Characteristics. It is an AE that meets the criteria of being related, serious, and unexpected. The term "unexpected" means that for an investigational drug, the event is not listed in the Investigator's Brochure, Package Insert, or Summary of Product Characteristics, or its nature and severity go beyond what is described in the Investigator's Brochure.

1. The sponsor is responsible for reporting SUSARs to all participating trial sites and ethics committees. For unexpected serious adverse reactions that are fatal or life-threatening, the sponsor must report them within 7 days of first becoming aware of the event and submit follow-up information within the subsequent 8 days. For non-fatal or non-life-threatening unexpected serious adverse reactions, the sponsor must report them within 15 days of first becoming aware of the event. The day the sponsor becomes aware of the event is considered Day 0.
2. If the trial sites and ethics committees can directly accept SUSAR reports from the sponsor, the sponsor must deliver one copy of the SUSAR report to the trial sites and ethics committees within the specified timeframe, and another copy, after being reviewed and signed by the investigator, must be submitted again to the trial sites and ethics committees.
3. If the trial sites and ethics committees only accept SUSAR reports reviewed by the investigator, the sponsor's delivery of the SUSAR and the investigator's review and sign-off must meet the regulatory timelines required for reporting to the trial sites and ethics committees.
4. When the sponsor and investigator cannot reach consensus on the causal relationship between the unexpected serious adverse event and the investig drugational, if either party cannot rule out a relationship with the investigational medication, the event should be reported promptly. The sponsor must rapidly report the SUSAR to all investigators and trial centers participating in the clinical trial, as well as to the ethics committees, drug regulatory authorities, and health administration departments.

### Reporting UPs to participants

### After the occurrence of an UP, proactive measures should be taken. Following the investigator's assessment of the relevance, timely communication with the patient is necessary to seek patient’s opinion on whether to continue participating in the study and receiving the intervention.

1. Statistical Analysis

## Analytical set

### Modified intention-to-treat (mITT) set/full analysis set (FAS)

### All subjects who are included in the mITT/FAS have received randomization and have at least two post-baseline efficacy assessments, regardless of whether they completed the entire treatment.

### Safety set (SS)

### All subjects who received at least one dose of the study drug after enrollment will be included in the safety analysis.

## Statistical analyse

### Sample size calculation

### Accroding to the results from the previous study on the perospirone combination therapy for patients with MDD, the response rate of the experimental group was approximately 78%~90%, and that of the placebo group was about 47%~71%. The maximum response rates of the two groups were used for the estimation of the sample size. Using Power Analysis and Sample Size Software 8.0, we conducted chi-square test to determine that a sample size of 174 participants (87 per group) would provide 80% power (β = 0.2) at a two-sided alpha level of 0.05 (α = 0.05) to detect a significant between-group difference in the primary efficacy outcome. Considering a 20% dropout rate, the final sample size was adjusted to 210 participants (105 per group).

### Efficacy end points analyse

### To make the results of the statistical analysis more precise, efficacy analyses are performed in the FAS. Missing data in the FAS will be imputed with the last-observation-carried-forward (LOCF) approach.

### Efficacy end points including:

**A.Primary efficacy end points:**

1. MADRS response rate: the proportion of participants with a ≥50% reduction in the MADRS score from baseline at week 4 and week 8.

2. MADRS remission rate: The proportion of participants with an MADRS score ≤10.

**B.Secondary efficacy end points:**

1. .Reduction rates in MADRS and Hamilton Anxiety Scale (HAMA) scores at each visit.

2. Change rates in each Clinical Global Impression (CGI) and Beck Suicidal Ideation Scale (BSI-CV) subscales scores at each visit.

3. Reduction rates in Pittsburgh Sleep Quality Index (PSQI), 16-item Quick Inventory of Depressive Symtomatology-Self-Report (QIDS-SR16), Generalized Anxiety Disorder 7-item Scale (GAD-7), and Snaith-Hamilton Pleasure Scale (SHAPS) scores at each visit.

4. Change rates in Sheehan Disability Scale (SDS) and Quality of Life Enjoyment Questionnaire - Short Form (Q-LES-Q-SF) scores at each visit.

5. Change rates in neurocognitive function test performance before and after treatment.

### Safety end points analyses

The safety set (SS), included of individuals who have received at least one dose of the randomized investigational drug, were employed to analyze the safety and tolerability profile of the medication by comparing the self-reported AEs/SAEs of the two groups of patients. Since the AEs covered by the TESS involve multiple systems, which could assess the safety of the drug in a more comprehensive manner. Therefore, the TESS was also used for safety evaluation. Nevertheless, since the TESS set to be completed by patients during follow-up visits, on the basis of the SS sample, those with at least one follow-up visit were included for inter-group comparisons of the incidence of AEs based on the TESS. For TESS items, a score of 0 indicates that the patient did not experience the corresponding AE. Conversely, any score greater than 0 signified that the patient experienced the AE during the treatment period.

### Statistical analysis methods

1. **Homogeneity Analysis:** Baseline comparison analysis of demographic and clinical characteristics of the participants enrolled in each group will be conducted to assess comparability between groups.
2. **Efficacy Analysis:** Statistical analyses of the primary and secondary efficacy end points will be performed based on the FAS for comparisons between groups.
3. **Safety Analysis:** The incidence of AEs and AEs related to the study drug will be compared between groups. A tabular description will be provided for AEs occurring during the trial, changes in laboratory test results (normal/abnormal) before and after the trial, and the relationship between abnormal changes and the study drug.
4. **Overall participant profile:** For the overall information of participants, a flowchart was used to describe the total number of screened individuals, reasons for screen failures, randomized participants, those who received treatment, and those who discontinued or were excluded, along with detailed explanations, and so on. Specific descriptions of screening, randomization and enrollment condition, together with the details of those who failed to pass the screening, were further reported in the following step. Finally, the overall completion and incomplete status of participants from the experimental and control group, and detailed information about each participant who did not complete the experiment were described respectively.
5. **Demographic and clinical characteristics of participants at baseline:** Baseline demographic and clinical characteristics was analyzed to assess comparability between the groups, beginning with statistical description. Continuous variables were summarized using means, medians, standard deviations, quartiles, and ranges. Normality was tested, followed by between-group comparisons via t-tests for normally distributed data or Wilcoxon rank-sum tests for nonparametric data. Categorical and ordinal variables will be expressed as frequencies and percentages, analyzed with chi-square or Fisher’s exact tests.
6. **Analysis of Primary Efficacy Outcomes**

Response Rate: The proportion of participants with a reduction of ≥ 50% in MADRS scores at Weeks 4 and 8.

Clinical Remission Rate: The proportion of participants with a MADRS score ≤ 10 at Weeks 4 and 8.

The difference of primary and secondary efficacy outcomes between the groups was calculated based on both FAS and PPS. For primary efficacy outcomes, including response rate and clinical remission rate, a logistic regression model was performed to analysis the differences between the Pipamperone + SSRIs/SNRIs group and the Placebo + SSRIs/SNRIs group at each follow-up time point, along with the placebo treatment as the control. Treatment group was included as a factor in the model, with baseline MADRS total score as a covariate.

1. **Analysis of Secondary Efficacy Outcomes:** For secondary efficacy outcomes, the changes from baseline to Week 8 were analyzed. A mixed model for repeated measures (MMRM) was used to compare the differences in secondary outcomes between the Pipamperone + SSRIs/SNRIs group and the Placebo + SSRIs/SNRIs group at each follow-up time point. Least square mean (LSM) changes in scores during the trial process were reported for both groups. In the MMRM, treatment group (i.e., placebo treatment as control group), visit time points, study center, baseline total scores of assessment scales, and the interaction term between treatment group and visit time points were included as fixed effects. Individual participants were considered as random effects. An unstructured covariance matrix was used for residuals to compare efficacy differences between treatment groups.

The secondary efficacy analyses included details as follows:

The improvement in alleviating anxiety symptoms was evaluated by comparing pre- and post-treatment changes in the Montgomery-Åsberg Depression Rating Scale (MADRS) and the Hamilton Anxiety Rating Scale (HAMA).

The improvement in quality of life and social functioning was evaluated by changes in the Sheehan Disability Scale (SDS) and Quality of Life Enjoyment Questionnaire - Short Form (Q-LES-Q-SF).

The improvement in alleviating other depressive symptoms was evaluated by the reduction rates in the Quick Inventory of Depressive Symptomatology-Self Report (QIDS-SR16) and the Generalized Anxiety Disorder-7 (GAD-7), evaluating the effects on anxiety, sleep disturbances, and anhedonia.

1. **Analysis of Safety Outcomes:** The safety of Pipamperone augmentation therapy for antidepressants in the treatment of depression, as well as the incidence of adverse events, was assessed based on the Treatment Emergent Symptom Scale (TESS). The TESS was used to measure common adverse reaction symptoms and signs, and selected laboratory test results, with severity ratings for each symptom.

For total TESS scores at each visit, normality was assessed. For normally distributed dataset, an independent t-test was conducted to compare differences between the groups; otherwise, the Wilcoxon rank-sum test was used. For each specific sub-score in the TESS, chi-square tests or Fisher’s exact tests were used to compare differences between groups.

The evaluation of adverse events included categorization, severity ratings, relationship with the drug, management measures and outcomes. The incidence of adverse events was calculated as the ratio of the number of participants experiencing adverse events to the number of participants eligible for safety evaluation. Chi-square tests or Fisher’s exact tests were conducted to compare the frequency, occurrence rate, and incidence of adverse events, serious adverse events, and adverse events related to the drug between groups. All adverse events observed in this trial were presented in the table.

All statistical analyses were performed using the Statistical Analysis System (SAS), and statistical significance was determined by a two-sided p value of <0.05, with a p value of <0.1 indicating a trend toward significance.

1. Supporting Documentation and Operational Considerations

## Regulatory, ethical, and study oversight considerations

### Informed consent process

Informed consent is a process that begins before an individual agrees to participate in a study and continues throughout their involvement in the research. The informed consent document will be approved by the Institutional Review Board (IRB), and participants will be asked to read and review it. The investigator will explain the study to the participant and answer any questions they may have. The investigator will orally explain to the participant, in a manner that they can understand, the purpose of the study, the procedures involved, potential risks, and the rights of the participant. Before signing the written informed consent, the participant should be given ample time to read carefully and ask questions. The participant should have the opportunity to discuss the study with family members or representatives or to reflect on it themselves before agreeing to participate. The participant will sign the informed consent document before any study-related procedures are conducted. The participant must be informed that participation in the study is voluntary and that they can withdraw from the study at any time without penalty. The investigator will provide the participant with a copy of the informed consent document for their records. The informed consent process should be conducted before the participant undergoes any study-related procedures, and the process (including the date) should be documented in the source documents, along with the retention of the signed informed consent form. The investigator must specifically inform the participant that "if they choose not to participate in this study, their quality of medical care will not be adversely affected," to ensure the rights and welfare of the participant.

### Suspension and termination of the study

If there are sufficient and reasonable grounds, this study may be temporarily suspended or prematurely terminated. The party deciding to suspend or terminate the study shall provide written notification to the participants, investigators, funding agencies, clinical trial sponsors, and regulatory authorities, and document the reasons for the suspension or termination of the study. If the study is prematurely terminated or suspended, the PI shall promptly notify the participants, the IRB, and the sponsor, and provide the reasons for the study termination or suspension. Where applicable, the investigator will contact the participants and inform them of changes to the visit schedule. Reasons for suspension or termination may include, but are not limited to:

1. Identification of unexpected, significant, or unacceptable risks to participants.
2. Non-compliance with the protocol requirements.
3. Incomplete data and/or data insufficient for evaluation.
4. Confirmation that the primary endpoint has been reached.
5. Determination that the study is futile.

The study may resume only after issues related to safety, protocol compliance, and data quality have been resolved and meet the requirements of the sponsor, the IRB, and/or the National Medical Products Administration (NMPA).

### Confidentiality and privacy

All investigators, staff, and sponsors involved in the study must strictly maintain confidentiality and protect the privacy rights of the participants. In addition to clinical information related to the participants, this confidentiality will extend to cover tests involving biological sample analysis and genetic testing. Therefore, the study protocol, documents, data, and all other information generated during the study will be kept strictly confidential. No study materials or data may be disclosed to unauthorized third parties without the written permission of the sponsor.

All research activities will be conducted in as private an environment as possible. Monitors of the study, other authorized representatives of the sponsor, representatives of the IRB, regulatory authorities, or representatives of the pharmaceutical company providing the study products may inspect all documents and records that the investigator is required to maintain, including but not limited to the medical records of the study participants (in offices, clinics, or hospitals) and pharmacy records. The clinical research center should allow access to these records. Contact information of the participants will be securely stored at each clinical research center for internal use during the study. At the end of the study, all records will continue to be kept at the sponsor's institution for five years after the publication of the results.

Participant study data used for statistical analysis and research reporting will be transferred and stored in the data management center. This does not include the participants' contact information or identifying details. Instead, individual participants and their study data will be identified through a unique study identification code. Research data entered by researchers at the clinical research center and the data management system used will be secured and password-protected. At the end of the study, all study databases will be de-identified and stored at the sponsor's institution.

### Future utilization of stored samples and data

The data collected in this study will be stored and analyzed in the data management center. Upon completion of the study, de-identified archival data will be transferred and stored by the sponsor for use by other researchers, including those outside this study. Permission to transfer data to other researchers will be included as part of the informed consent process.

With the consent of the participants and approval from the local IRB, biological samples that do not contain personal information will be stored at each participating center, with the same purpose as the shared data repository maintained by the sponsor. While maintaining the anonymity of participants' personal information, a code linking the stored biological samples to the phenotypic data of the source participants will be provided to each center.

During the course of the study, participants may choose to withdraw their consent for storing their biological samples for future research. However, after the study is completed, consent for the storage of biological samples may not be withdrawn.

Upon completion of the study, access to the study data and/or samples will be provided through the sponsor.

### Clinical trial monitoring

This study will be subject to regular on-site monitoring visits by clinical monitors designated by the sponsor at the study hospitals. These visits are designed to ensure that the rights of participants in the clinical observations are safeguarded, that the data in the study records and reports are accurate and complete, and that the study adheres to the approved protocol, Good Clinical Practice (GCP) guidelines, and relevant regulations. This ensures that all aspects of the study protocol are strictly followed and that study documentation is accurately completed.

Investigators should truthfully, meticulously, and diligently record all items in the CRF in accordance with the requirements for completing the CRF, to ensure that the content of the CRF is genuine and reliable.

All observations and findings in the clinical observations should be verified to ensure the reliability of the data and to confirm that all conclusions drawn from the clinical observations are derived from the original data. Corresponding data management measures are in place during both the clinical observation and data processing stages.

### Quality control (QC)

Each research center will implement quality management of the study process, data and biological sample collection, and documentation archiving and study completion. Personalized quality management plans will be developed for each institution.

Quality control (QC) begins with the data entry system, and data QC checks will be run on the generated database. Any missing or abnormal data identified will be communicated to the trial institution for clarification or determination.

Monitors will verify, in accordance with written standard operating procedures (SOPs), whether the study is conducted in compliance with the protocol, International Council for Harmonisation of Technical Requirements for Pharmaceuticals for Human Use Good Clinical Practice (ICH GCP), and relevant applicable regulations [e.g., Good Laboratory Practice (GLP), Good Manufacturing Practice (GMP)], including the trial process, generation/ collection of data and biological samples, archiving (record-keeping), and reporting.

Clinical trial institutions will provide direct access to all study-related units, source data/documents, and reports for activities such as sponsor monitoring, auditing, or inspection by local regulatory authorities.

### Management of data and records collection and retention

Each participating institution shall comply with ICH GCP and relevant laws and regulations regarding the protection of participants' privacy and retain applicable medical and research records. Institutions should permit authorized representatives of the funder, sponsor, and regulatory authorities to inspect clinical records for quality assurance purposes (including photocopying as permitted by law), conduct reviews or audits, and evaluate the study's safety, progress, and data validity.

- - - 1. Responsibilities for data collection and management

Trial staff are responsible for data collection. Investigators should oversee their work and ensure the accuracy, completeness, legibility, and timeliness of the reported data. All source documents should be kept clear and tidy to ensure that the data can be accurately identified. The permanent copies of study visit records will be considered source documents for recording data of enrolled participants. Data entered into the electronic Case Report Forms (eCRFs) should be derived from the source documents and be consistent with the source data.

Clinical data [including AEs, concomitant medications, and data on expected drug AEs] and laboratory data will be entered into the database, which is provided by the data management center and complies with national regulations. The data system includes password protection and internal quality check functions, such as automatic range checks that can identify inconsistent, incomplete, or inaccurate data. Clinical data will be entered directly based on the source documents.

- - - 1. Retention of research data and records

The data of this study will be retained at the sponsor's institution for five years after the publication of the study results

### Protocol violation

Protocol violation refers to any non-compliance with the clinical trial protocol, the ICH GCP, or the Manual of Procedures (MOP). Non-compliance may arise from participants, investigators, or study center staff. In the event of deviations, corrective actions should be taken and completed promptly.

Investigators at the study centers are responsible for being vigilant about protocol violations. Upon identifying a violation, the investigator must report it in accordance with the Standard Operating Procedures (SOPs) for handling protocol deviations established by the local IRB and the clinical trial institution. All protocol violations must be documented in the source documents and submitted for review as required by the IRB. It is the investigator's responsibility to be aware of and comply with the relevant regulations of the IRB.

### Publication and Data Sharing Policy

After the completion of the study, the lead institution has the right to publish the summary report of this clinical observation in the form of a paper. Investigators from each participating institution and the sponsor are entitled to co-authorship of the paper.

### Conflict of interest policy

The study should not be influenced by any existing or foreseeable factors (such as pharmaceutical companies). Therefore, any individuals involved in the design, conduct, analysis, publication, or other aspects of this study will disclose and manage any actual conflicts of interest related to the study. Moreover, during the design and implementation of the study, individuals who may have conflicts of interest will be required to control such situations through appropriate methods.

## Other considerations

Eligibility and Exclusion Criteria Explanation:

1. Inclusion Criterion 2: "The current episode duration is within one year," which is calculated from the date of entry into the screening phase.
2. Inclusion Criterion 3: "Failed to respond adequately to at least one kind of antidepressant at therapeutic doses for at least 4 weeks" is defined as:

a) A reduction rate of less than 50% in the Hamilton Depression Rating Scale (HDRS) or the Montgomery-Åsberg Depression Rating Scale (MADRS) scores after four weeks of antidepressant use;

b) Participants must have been on antidepressant treatment for more than four weeks before enrollment, with the MADRS score at baseline screening ≥20 points and must meet Inclusion Criterion 1.

3. Exclusion Criterion 3: "Participants who have used antidepressants and/or antipsychotics during the current episode (low-dose benzodiazepines are permitted)" defines "low-dose" as: The dose does not exceed the conventional dosage for the indication of depression.

4. Exclusion Criterion 10: "Participants receiving hormonal therapy" refers to: hormonal medications, including those administered orally, by intramuscular injection, or intravenously.

1. References
2. WHO. The global burden of disease: 2004 update. Geneva: World Health Organization, 2008.
3. Huang YQ, Wang Y, Wang H, et al. Prevalence of Mental Disorders in China: a Cross-sectional Epidemiological Study. Lancet Psychiatry, 2019, 6: 211-224.
4. Onrust SV, McClellan K. Perospirone. CNS Drugs, 2001, 15(4): 329-37.
5. Yoshino T, Nisijima K, Shioda K, et al. Perospirone, a novel atypical antipsychotic drug, potentiates fluoxetine-induced increases in dopamine levels via multireceptor actions in the rat medial prefrontal cortex. Neuroscience Letters, 2004, 364(1): 16-21.
6. Shiwa T, Amano T, Matsubayashi H, et al. Perospirone, a novel antipsychotic agent, hyperpolarizes rat dorsal raphe neurons via 5-HT1A receptor. Journal of Pharmacological Sciences, 2003, 93(1): 114-7.
7. Schmidt AW, Lebel LA, Howard Jr HR, et al. Ziprasidone: a novel antipsychotic agent with a unique human receptor binding profile. European Journal of Pharmacology, 2001, 425(3): 197-201.
8. Dunner DL, Amsterdam JD, Shelton RC, et al. Efficacy and tolerability of adjunctive ziprasidone in treatment-resistant depression: a randomized, open-label, pilot study. Journal of Clinical Psychiatry, 2007, 68(7): 1071-7.
9. Sato S, Mizukami K, Moro K, et al. Beneficial effects of perospirone on aggressive behavior associated with dementia. Psychiatry and Clinical Neurosciences, 2006, 60(1): 106-9.
10. Ojima T, Ito C, Sakurai E, et al. Effects of serotonin-dopamine antagonists on prepulse inhibition and neurotransmitter contents in the rat cortex. Neuroscience Letters, 2004, 366(2): 130-4.
11. Kato T, Hirose A, Ohno Y, et al. Binding profile of SM-9018, a novel antipsychotic candidate. Japanese Journal of Pharmacology, 1990, 54(4): 478-81.
12. Araki T, Yamasue H, Sumiyoshi T, et al. Perospirone in the treatment of schizophrenia: effect on verbal memory organization. Psychiatry Research, 2009, 172(3): 180-3.
13. Mori K, Nagao M, Yamashita H, et al. Effect of switching to atypical antipsychotics on memory in patients with chronic schizophrenia. Progress in Neuro-Psychopharmacology & Biological Psychiatry, 2004, 28(4): 659-65.
14. Sumiyoshi T, Higuchi Y, Itoh T, et al. Effect of perospirone on P300 electrophysiological activity and social cognition in schizophrenia: a three-dimensional analysis with sloreta. Psychiatry Research, 2009, 172(3): 180-3.
15. Ohno Y. Pharmacological characteristics of perospirone hydrochloride, a novel antipsychotic agent. Nihon Yakurigaku Zasshi Folia Pharmacologica Japonica, 2000, 116(4): 225-31.
16. Yamashita H, Mori K, Nagao M, et al. Influence of aging on the improvement of subjective sleep quality by atypical antipsychotic drugs in patients with schizophrenia: comparison of middle-aged and older adults. The American Journal of Geriatric Psychiatry, 2005, 13(5): 377-84.Togo T, Iseki E, Shoji M, Oyama I, et al. Prolactin levels in schizophrenic patients receiving perospirone in comparison to risperidone. Journal of Clinical Psychiatry, 2005, 66(11): 1464-7.
17. Protocol Adjustments During the Implementation of the Trial

Due to the challenges encountered during the actual implementation of the trial, we have made appropriate adjustments based on the original protocol:

1. **Adjustments to participating centers**: due to the slow enrollment rate at the originally planned number of centers (7 centers), the number of centers has been expanded to 10 centers for competitive enrollment, including Beijing HuiLongGuan Hospital (Beijing, China), Hebei Provincial Mental Health Center (Shijiazhuang, Hebei, China), Hunan Brain Hospital (Changsha, Hunan, China), Nanjing Brain Hospital (Nanjing, Jiangsu, China), Xiamen XianYue Hospital (Xiamen, Fujian, China), Shanxi Provincial Mental Health Center (Taiyuan, Shanxi, China), The First Affiliated Hospital of Zhejiang University (Hangzhou, Zhejiang, China), Xiangya Second Hospital of Central South University (Changsha, Hunan, China), The First Affiliated Hospital of Chongqing Medical University (Chongqing, China), The Fifth People's Hospital of Zigong City (Zigong, Sichuan, China).

**2. Adjustments to the dosing regimen:** The initial dosing regimen was based on the approved product label for schizophrenia, as no evidence-based guidelines for perospirone existed in MDD at the time of study design. However, due to differences in symptom profile, treatment goals, and anticipated tolerability between schizophrenia and MDD, the protocol was pragmatically adapted in clinical practice. Dosing was typically initiated at 4–8 mg/day (or <4 mg/day in older or milder patients), with titration guided by symptom severity and tolerability during the first two weeks; doses were generally stabilized by Week 2. The maintenance dose ranged from 4 to 24 mg/day and was administered once or twice daily. Higher doses (>24 mg/day) were used on a case-by-case basis for patients with more severe symptoms, always within the approved maximum of 48 mg/day. All adjustments were made under the supervision of experienced clinicians.

**3. Adjustments to sample size calculation:** The sample size was finally calculated based on previous studies of SGA adjunctive therapy in patients with MDD, which reported response rates ranging from 10.5% to 58.9% in the SGA group and from 6.8% to 46.3% in the placebo group. The pair of groups showing the largest difference in response rates (46.6% vs. 26.6%) was selected as the basis for sample size estimation. A Chi-square test was performed using Power Analysis and Sample Size (PASS) software, version 21.0.3, to determine the required sample size. With a two-sided alpha level of 0.05 and 80% power (β = 0.2), a total sample size of 176 participants (88 per group) was estimated to detect a statistically significant difference in the primary efficacy outcome. To account for an anticipated dropout rate of 10%, the initial sample size was increased to 196 participants (98 per group). Given the possibility of a higher-than-expected dropout rate, the final sample size was further adjusted to 210 participants (105 per group), which also provides sufficient power to detect a significant difference in remission rates (36.8% vs. 18.9%) under the same statistical assumptions.

**4. Adjustments to efficacy end points**: the primary efficacy end points were the MADRS response rate (defined as an improvement of ≥50% in the MADRS score from baseline) and remission rate (defined as a MADRS score of ≤10) at the end of treatment (week 8). The secondary efficacy end points were the response and remission rates at week 4, and the scores reductions of the scales mentioned in the protocol at each visit point.

**5. Adjustments to statistical methods**: Efficacy analyses were performed in the FAS. Prior to initiating the analysis of efficacy outcomes, Breslow-Day test was carried out to assessed whether there was any centre effect that contributed to the results in the multi-centre trial.

For the primary efficacy outcomes and partial secondary efficacy outcomes (i.e. response and remission rates at weeks 4 and weeks 8), last observation carried forward (LOCF) method was used to handle the missing data in the anaysis of these outcomes. Since these outcomes were defined based on MADRS scores at the corresponding visit (response: ≥50% reduction from baseline; remission: MADRS score ≤10), missing MADRS values in the FAS were imputed using LOCF, and response or remission status was subsequently determined based on the imputed scores. Logistic regression models were employed to analyze and compare these outcomes between the the perospirone + SSRIs/SNRIs group (perospirone group) and the placebo + SSRIs/SNRIs group (placebo group), using results of the placebo group as reference. In the logistic regression model, treatment group was included as factor, and baseline MADRS total score was included as a covariate.

For the other secondary efficacy measures, the mean change of the included scales (MADRS, QIDS-SR16, HAMA, and Q-LES-Q-SF) from the baseline (Week 0) to Week 4 and Week 8 were analyzed. The mean changes and the differences of the mean changes of the secondary efficacy outcomes between the perospirone group and the placebo group were calculated and compared through the use of a mixed model for repeated measures (MMRM) analysis (with an unstructured covariance matrix). This method is widely recommended for longitudinal data with missing values under the missing at random (MAR) assumption, as it utilizes all available data and does not require explicit imputation. The model included the baseline total scores of each scales, study centre (centre 1 to centre 10), treatment group (perospirone and placebo), time (week 2, week 4 and week 8), and time-by-treatment interaction as fixed effects, with the subjects-specific effects as a random effect. The least squares mean (LSM) changes of the scores for the two groups were reported.

Chi-square tests or Fisher’s precision probability tests were conducted to compare the overall incidence rates of AEs, SAEs and AEs leading to treatment discontinuation, as welll as the incidence rates of each drug related-AE between the two groups. All statistical analyses were done in Statistical Analysis System (SAS) version 9.4. Statistical significance was determined by a two-sided p value less than 0.05
