# Supplementary material for: Efficacy and safety of perospirone as adjunctive therapy in major depressive disorder patients with inadequate response to antidepressants: a randomized clinical trial
Source: eClinicalMedicine. 2025 Nov 7;90:103626. doi: 10.1016/j.eclinm.2025.103626 (PMC12766416; doi:10.1016/j.eclinm.2025.103626)
Supplement: Supplementary Tables [file mmc2.docx]

**Supplementary Material
Contents:**

**1.Supplementary methods**

1.1. Statistical analysis: logistic regression model diagnostics

1.2. Statistical analysis: logistic formal interaction tests in subgroup analyses

2. Supplementary Table S1: Diagnostic checks for logistic regression model assumptions for response and remission outcomes at 4 and 8 weeks.

3. Supplementary Table S2. Descriptive analysis of medication dosage utilization.

4.Supplementary Table S3. Primary and secondary efficacy outcomes (response and remission rates) in the per-protocol set (PPS).

5. Supplementary Table S4. Primary and secondary efficacy outcomes (response and remission rates) using multiple imputation in the full analysis set (FAS).

6. Supplementary Table S5. Comparison of primary and sensitivity analyses for efficacy outcomes (response and remission rates).

7. Supplementary Figure S1. Key secondary efficacy end points (response rate and remission rate at week 4).

8. Supplementary Table S6. Secondary efficacy outcomes (changes in rating scale scores) in the per-protocol set (PPS).

9. Supplementary Table S7. Secondary efficacy outcomes (changes in rating scale scores) in the full analysis set (FAS) using multiple imputation.

10. Supplementary Table S8. Comparison of primary and sensitivity analyses for efficacy outcomes (changes in rating scale scores).

11. Supplementary Table S9. Adverse event and serious adverse event analysis.

12. Supplementary Table S10. The incidence of ddverse events potentially related to the investigational drug (reported by patients).

13. Supplementary Table S11. All adverse events that occurred during the trial period.

14. Supplementary Table S12. Demographic and clinical characteristics of participants in the 4-Week and 4–8-Week subgroups at baseline.

15. Supplementary Table S13. Efficacy outcomes (response and remission rates) in the 4-Week subgroup.

16. Supplementary Table S14. Efficacy outcomes (response and remission rates) in the 4–8-Week subgroup.
17. Supplementary Table S15. Comparison of treatment effects on response and remission between the 4-Week and 4–8-Week subgroups.

18. Supplementary Table S16. Tests of treatment-by-subgroup interaction for the primary efficacy outcomes.

19. Supplementary Table S17. Secondary efficacy outcomes (changes in rating scale scores) in the 4-Week subgroup.

20. Supplementary Table S18. Secondary efficacy outcomes (changes in rating scale scores) in the 4–8-Week subgroup.

21. Supplementary Table S19. Comparison of treatment effects on secondary efficacy outcomes (changes in rating scale scores) between the 4-Week and 4–8-Week subgroups.

22. Data Sharing Statement

**1. Supplementary methods**

**1.1. Statistical analysis: logistic regression model diagnostics**

To assess the validity of the logistic regression models used in the analysis of binary efficacy outcomes (response and remission at 4 and 8 weeks), several diagnostic checks were performed using IBM SPSS Statistics 25·0. The procedures are described below.

Multicollinearity was evaluated by using variance inflation factors (VIFs). VIFs were computed by fitting a linear regression model with the same set of predictors (treatment group and baseline MADRS score) and enabling the "Collinearity Diagnostics" option (Analyze > Regression > Linear). A VIF < 3 is generally considered to indicate no substantial multicollinearity.

The assumption of linearity between the continuous predictor (baseline MADRS scores) and the logit was examined through the Box-Tidwell procedure. The natural logarithm of baseline MADRS score was first computed (Transform > Compute Variable: ln_base = LN(baseline_MADRS score)), and an interaction term between the original variable and its log was created (int_base_ln = baseline_MADRS scores × ln_base). This interaction was then added to each logistic regression model. A non-significant result (p > 0·05) was taken as evidence that the linearity assumption was satisfied.

Model calibration was assessed using the Hosmer-Lemeshow goodness-of-fit test, which is automatically generated by SPSS during binary logistic regression (Analyze > Regression > Binary Logistic). The test compares observed and expected event frequencies across deciles of predicted risk. A p > 0·05 is typically interpreted as no significant lack of fit, indicating acceptable model calibration.

The adequacy of sample size was evaluated using the events-per-variable (EPV) ratio, calculated as the number of outcome events divided by the number of predictor variables in each model. Event counts were obtained from frequency distributions (Analyze > Descriptive Statistics > Frequencies). An EPV ratio of at least 10 was considered sufficient to support stable estimation and reduce the risk of overfitting.

**1.2. Statistical analysis: formal interaction tests in subgroup analyses**

To examine whether the effect of adjunctive perospirone differed across patient subgroups, formal tests of interaction were conducted using logistic regression. Subgroups were defined based on the duration of antidepressant treatment in the current episode (exactly 4 weeks vs. 4–8 weeks). A multiplicative interaction term between treatment group (perospirone vs. placebo) and subgroup was included in the model. The analysis was adjusted for baseline MADRS score, and the model contained the main effects of treatment, subgroup, baseline MADRS, and the treatment-by-subgroup interaction term. The primary efficacy outcomes (including response and remission at week 8) were analyzed using the Wald test, with a two-sided p-value < 0.05 considered statistically significant for the interaction term. A statistically significant interaction (p < 0.05) would indicate that the magnitude of the treatment effect differs significantly between subgroups, suggesting that the treatment effect may be related to when perospirone is added in patients with inadequate response.

**2 . Supplementary Table S1. Diagnostic checks for logistic regression model assumptions for response and remission outcomes at 4 and 8 weeks.**

| **Logistic models** | **VIF** | **HOSMER-LEMESHOW**  **P value** | **BOX-TIDWELL P value (INT BASELINE)** | **EPV ratio** |
| --- | --- | --- | --- | --- |
| 8 weeks response | 1·000 | 0·83 | 0·70 | 56·5 |
| 8 weeks remission | 1·000 | 0·12 | 0·79 | 41·5 |
| 4weeks response | 1·000 | 0·29 | 0·74 | 40·0 |
| 4 weeks remission | 1·000 | 0·054 | 0·18 | 26·5 |

A VIF < 3 indicates no multicollinearity; a Hosmer-Lemeshow test *p* > 0·05 indicates acceptable model calibration; a non-significant Box-Tidwell interaction (p > 0·05) supports the assumption of linearity between the continuous predictor and the logit; and an EPV ≥ 10 is considered adequate for stable model estimation.

Abbreviation: VIF,variance inflation factor; EPV, events per variable.

**3. Supplementary Table S2. Descriptive analysis of medication dosage utilization.**

| **Visit point** | **Medication dosage (mg/d)** | **Number of patients (N=)** | **Proportions of patients (%)** |
| --- | --- | --- | --- |
| Baseline  (Week 0 -2 days) | <4 | 3 | 1·69 |
|  | 4 | 83 | 46·89 |
|  | 8 | 85 | 48·02 |
|  | 12 | 5 | 2·82 |
|  | 16 | 1 | 0·56 |
|  | Total | 177 | 100·00 |
| Visit 1  (Week 2±2 days) | <4 | 2 | 1·13 |
|  | 4 | 43 | 24·43 |
|  | 6 | 15 | 8·52 |
|  | 8 | 78 | 44·32 |
|  | 12 | 22 | 12·50 |
|  | 16 | 13 | 7·39 |
|  | 20 | 1 | 0·57 |
|  | 24 | 2 | 1·13 |
|  | Missing data | 1 | - |
|  | Total | 177 | 100·00 |
| Visit 2  (Week 4±2 days) | <4 | 2 | 1·13 |
|  | 4 | 40 | 22·73 |
|  | 6 | 11 | 6·25 |
|  | 8 | 82 | 46·59 |
|  | 12 | 16 | 9·09 |
|  | 16 | 20 | 11·36 |
|  | 20 | 1 | 0·57 |
|  | 24 | 4 | 2·27 |
|  | Missing data | 1 | - |
|  | Total | 177 | 100·00 |
| Visit 3  (Week 8±2 days) | <4 | 3 | 1·91 |
|  | 4 | 35 | 22·73 |
|  | 6 | 8 | 5·19 |
|  | 8 | 70 | 45·45 |
|  | 12 | 16 | 10·39 |
|  | 16 | 18 | 11·69 |
|  | 20 | 1 | 0·65 |
|  | 24 | 2 | 1·30 |
|  | >24 | 1 | 0·65 |
|  | Missing data | 23 | - |
|  | Total | 177 | 100·00 |

Percentages were calculated based on the number of patients with available dose data at each time point.

**4.****Supplementary Table S3. Primary and secondary efficacy outcomes (response and remission rates) in the per-protocol set (PPS).**

| **Outcomes** | **Perospirone group (n=78)** | **Placebo group (n=80)** | **Odds ratio (95% CI)** | **P value** |
| --- | --- | --- | --- | --- |
| **Primary efficacy outcomes** | | | | |
| Response at Week 8, no·(%) | 55 (70·5) | 52 (65·0) | 1·315 (0·670-2·579) | 0·43 |
| Remission at Week 8, no·(%) | 44 (56·4) | 34 (42·5) | 1·671 (0·883-3·162) | 0·12 |
| **Secondary efficacy outcomes** | | | | |
| Response at Week 4, no·(%) | 44 (56·4) | 30 (37·5) | 2·238 (1·176-4·261) | 0·014 |
| Remission at Week 4, no·(%) | 31 (39·7) | 17(21·3) | 2·344 (1·154-4·759) | 0·018 |

Per-protocol set (PPS) includes all participants who adhered to the study protocol, completed the treatment period, and had no major protocol violations (e.g., non-compliance, incorrect dosing, or unauthorized medications).

**5. Supplementary Table S4. Primary and secondary efficacy outcomes (response and remission rates) using multiple imputation in the full analysis set (FAS).**

| **Outcomes** | **Perospirone group (n=87)** | **Placebo group (n=90)** | **Odds ratio (95% CI)** | | **P value** |
| --- | --- | --- | --- | --- | --- |
| **Primary efficacy outcomes** | | | | | |
| Response at Week 8, no·(%) | 59·4 (68·3) | 56 (62·2) | 1·309 (0·670-2·557) | 0·43 | |
| Remission at Week 8, no·(%) | 46 (52·9) | 36·7 (40·8) | 1·635 (0·877-3·049) | 0·12 | |
| **Secondary efficacy outcomes** | | | | | |
| Response at Week 4, no·(%) | 48 (55·2) | 32 (35·6) | 2·242 (1·224-4·107) | 0·0090 | |
| Remission at Week 4, no·(%) | 35 (40·2) | 18(20·0) | 2·701 (1·373-5·313) | 0·0040 | |

Missing MADRS scores at follow-up visits were imputed using multiple imputation by chained equations (MICE, also known as fully conditional specification). Twenty imputations were generated using predictive mean matching (PMM), with baseline MADRS scores, treatment group, visit time, and the group-by-visit interaction as predictors. A lower bound of 0 was applied to prevent implausible negative values. Study ID and site were excluded from the model. The low overall missing rate (<10%) and balanced distribution across groups support the assumption of missing at random (MAR). Response and remission rates were calculated from imputed MADRS scores in each imputed dataset and combined using Rubin’s rules; therefore, the reported numbers are averaged across imputations and may not be integers.

**6. Supplementary Table S5. Comparison of primary and sensitivity analyses for efficacy outcomes (response and remission rates).**

|  | **Week 8** | | **Week 4** | |
| --- | --- | --- | --- | --- |
|  | **Response (OR)** | **Remission (OR)** | **Response (OR)** | **Remission (OR)** |
| **Primary analyses** | | | | |
| LOCF imputation in the FAS population | 1·406 | 1·944* | 2·243* | 2·701* |
| **Sensitivity analyses** | | | | |
| Multiple imputation in the FAS population | 1·309 | 1·635 | 2·242* | 2·701* |
| PPS population | 1·315 | 1·671 | 2·238* | 2·344* |

*: P < 0·05, indicating statistical significance at the 5% level.

Abbreviation: FAS, full analysis set; LOCF, last observation carried forward; OR, odds ratio; PPS, per-protocol set.


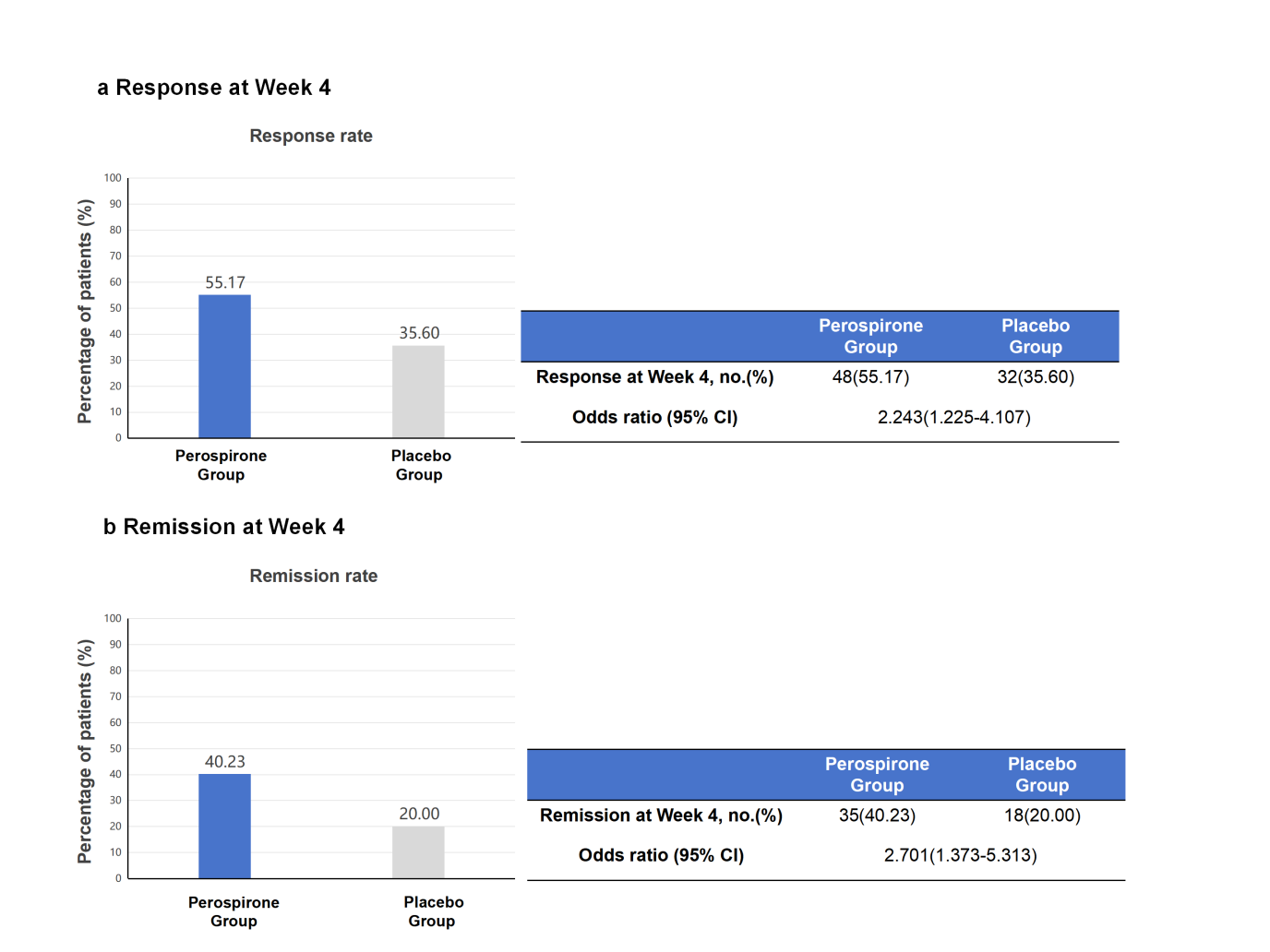


**7. Supplementary Figure S1. Key secondary efficacy end points (response rate and remission rate at week 4).**

**8. Supplementary Table S6. Secondary efficacy outcomes (changes in rating scale scores) in the per-protocol set (PPS).**

|  | **Week 4** | | |  | **Week 8** | | |  |
| --- | --- | --- | --- | --- | --- | --- | --- | --- |
|  | **Perospirone**  **group (n=78)** | **Placebo**  **group (n=80)** | **LSMD, vs.**  **placebo group**  **(95%CI)** | **P value** | **Perospirone**  **group (n=78)** | **Placebo**  **group (n=80)** | **LSMD, vs.**  **placebo group**  **(95%CI)** | **P value** |
|  | **LS mean (SE)** | **LS mean (SE)** |  |  | **LS mean (SE)** | **LS mean (SE)** |  |  |
| **Key secondary efficacy end points** | | | | | | | | |
| MADRS score | -14·48 (0·92) | -10·63 (0·92) | - 3·84(-6·24, -1·44) | 0·0019 | -17·19 (0·93) | -15·73 (0·93) | -1·46 (-3·91, 0·99) | 0·24 |
| QIDS-SR16 score | -6·43 (0·53) | -4·14 (0·53) | -2·29 (-3·67, -0·92) | 0·0012 | -6·45(0·59) | -5·95 (0·59) | -0·49 (-2·05, 1·07) | 0·53 |
| **Other secondary efficacy end points** | | | | | | | | |
| HAMA score | -8·54 (0·76) | -6·98 (0·77) | -1·55 (-3·55, 0·45) | 0·13 | -10·54 (0·71) | -8·98 (0·72) | -1·55 (-3·40, 0·30) | 0·099 |
| Q-LES-Q-SF score | 4·63 (0·94) | 2·41 (0·96) | 2·22 (-0·19, 4·64) | 0·071 | 6·59 (1·05) | 4·97 (1·07) | 1·62 (-1·13, 4·38) | 0·24 |

Abbreviation: HAMA, Hamilton Anxiety Scale; LS, least squares; LSMD, least squares mean difference; MADRS, Montgomery-Åsberg Depression Rating Scale; PPS, per-protocol set; QIDS-SR16, 16-item Quick Inventory of Depressive Symptomatology (Self-Report); Q-LES-Q-SF, Quality of Life Enjoyment and Satisfaction Questionnaire - Short Form; SE, standard error

**9. Supplementary Table S7. Secondary efficacy outcomes (changes in rating scale scores) in the full analysis set (FAS) using multiple imputation.**

|  | **Week 4** | | |  | **Week 8** | | |  |
| --- | --- | --- | --- | --- | --- | --- | --- | --- |
|  | **Perospirone**  **group (n=87)** | **Placebo**  **group (n=90)** | **LSMD, vs.**  **placebo group**  **(95%CI)** | **P value** | **Perospirone**  **group (n=87)** | **Placebo**  **group (n=90)** | **LSMD, vs.**  **placebo group**  **(95%CI)** | **P value** |
|  | **LS mean (SE)** | **LS mean (SE)** |  |  | **LS mean (SE)** | **LS mean (SE)** |  |  |
| **Key secondary efficacy end points** | | | | | | | | |
| MADRS score | -14·63 (0·87) | -10·25 (0·87) | -4·41 (-6·67, -2·15) | 0·00010 | -17·29 (0·90) | -15·46 (0·90) | -1·63 (-4·01, 0·76) | 0·18 |
| QIDS-SR16 score | -6·78 (0·51) | -4·13 (0·51) | -2·62 (-3·94, -1·30) | < 0·0001 | -6·75 (0·59) | -5·89 (0·58) | -0·79 (-2·29, 0·71) | 0·30 |
| **Other secondary efficacy end points** | | | | | | | | |
| HAMA score | -8·71 (0·73) | -6·54 (0·74) | -2·17 (-4·08, -0·27) | 0·026 | -10·62 (0·70) | -8·77 (0·70) | -1·69 (-3·55, 0·16) | 0·074 |
| Q-LES-Q-SF score | 4·97 (0·88) | 2·33 (0·89) | 2·67 (0·44, 4·91) | 0·019 | 6·96 (1·02) | 4·79 (1·03) | 1·97 (-0·66, 4·60) | 0·14 |

Missing scales scores at follow-up visits were imputed using multiple imputation by chained equations (MICE, also known as fully conditional specification). Twenty imputations were generated using predictive mean matching (PMM), with baseline scales scores, treatment group, visit time, and the group-by-visit interaction as predictors· A lower bound of 0 was applied to prevent implausible negative values. Study ID and site were excluded from the model. The low overall missing rate (<10%) and balanced distribution across groups support the assumption of missing at random (MAR). Least squares means (LSMs) and least squares mean differences (LSMDs) were estimated separately for each imputed dataset using a mixed model for repeated measures (MMRM) model. The final LSMs and LSMDs were combined across imputations using Rubin’s rules. All analyses were conducted using SAS 9.4 with PROC MIXED and PROC MIANALYZE.

Abbreviation: FAS, full analysis set; HAMA, Hamilton Anxiety Scale; LS, least squares; LSMD, least squares mean difference; MADRS, Montgomery-Åsberg Depression Rating Scale; QIDS-SR16, 16-item Quick Inventory of Depressive Symptomatology (Self-Report); Q-LES-Q-SF, Quality of Life Enjoyment and Satisfaction Questionnaire - Short Form; SE, standard error.

**10. Supplementary Table S8. Comparison of primary and sensitivity analyses for efficacy outcomes (changes in rating scale scores).**

|  | **Week 8** | | | | **Week 4** | | | |
| --- | --- | --- | --- | --- | --- | --- | --- | --- |
|  | **MADRS (LSMD)** | **QIDS-SR16 (LSMD)** | **HAMA (LSMD)** | **Q-LES-Q-SF (LSMD)** | **MADRS (LSMD)** | **QIDS-SR16 (LSMD)** | **HAMA (LSMD)** | **Q-LES-Q-SF (LSMD)** |
| **Primary analyses** | | | | | | | | |
| FAS population | -1·83 | -0·85 | -1·86* | 2·17 | -4·37* | -2·65* | -2·16* | 2·64* |
| **Sensitivity analyses** | | | | | | | | |
| Multiple imputation in the FAS population | -1·63 | -0·79 | -1·69 | 1·97 | -4·41* | -2·62* | -2·17* | 2·67* |
| PPS population | -1·46 | -0·49 | -1·55 | 1·62 | -3·84* | -2·29* | -1·55 | 2·22 |

The primary analyses was counduced in the full analysis set (FAS) using mixed-effects model for repeated measures (MMRM) method, which used all available data under the assumption that missing data were missing at random (MAR) and did not require imputation.

*: P < 0·05, indicating statistical significance at the 5% level.

Abbrevation: FAS, full analysis set; HAMA, Hamilton Anxiety Scale; LSMD, least squares mean difference; MADRS, Montgomery-Åsberg Depression Rating Scale; PPS, per-protocol set; QIDS-SR16, 16-item Quick Inventory of Depressive Symptomatology (Self-Report); Q-LES-Q-SF, Quality of Life Enjoyment and Satisfaction Questionnaire - Short Form;

**11. Supplementary Table S9. Adverse event and serious adverse event analysis.**

|  | **Perospirone group (n=108)** | **Placebo group (n=102)** | **P value** |
| --- | --- | --- | --- |
| AEs, number of events | 133 | 125 | - |
| SAEs, number of events (%) | 3 (2·26) | 4 (3·20) | 0·72 |
| Severity of AEs  Grade 1  Grade 2  Grade 3  Grade 4 | 96 (72·18)  33 (24·81)  1(0·75)  3 (2·26) | 97 (77·60)  24 (19·20)  1 (0·80)  3 (2·40) | 0·77 |
| Relationship to the investigational drug  Unrelated  Unlikely  Possible  Probable  Certain | 8 (6·02)  9 (6·77)  52 (39·10)  58 (43·61)  6 (4·51) | 13 (10·40)  4 (3·20)  50 (40·00)  58 (46·40)  0 (0·00) | 0·058 |
| Event status  Ongoing  Resolved | 25 (18·80)  108 (81·20) | 19 (15·20)  106 (84·80) | 0·44 |

Grade refers to the severity of an adverse event (AE), as defined by the National Cancer Institute (NCI) Common Terminology Criteria for Adverse Events (CTCAE), version 5.0. The grading scale is as follows: Grade 1 Mild; asymptomatic or mild symptoms; clinical or diagnostic observations only; intervention not indicated. Grade 2 Moderate; minimal, local or non-invasive intervention indicated; limiting age-appropriate instrumental activities of daily living (ADL). Grade 3 Severe or medically significant but not immediately life-threatening; hospitalization or prolongation of hospitalization indicated; disabling; limiting self care ADL. Grade 4 Life-threatening consequences; urgent intervention indicated. Grade 5 Death related to AE.

**12. Supplementary Table S10. The incidence of ddverse events potentially related to the investigational drug (reported by patients).**

| **Adverse events** | **Perospirone group**  **(N=108)** | **Placebo group**  **(N=102)** | **P value** |
| --- | --- | --- | --- |
|  | **Number of patients (%)** | |  |
| Somnolence | 8 (7·41) | 9 (8·82) | 0·71 |
| Dizziness | 6 (5·56) | 3 (2·94) | 0·55 |
| Nausea | 4 (3·70) | 4 (3·92) | 1·0 |
| Tiredness | 3 (2·78) | 2 (1·96) | 1·0 |
| Headache | 3 (2·78) | 2 (1·96) | 1·0 |
| Palpitation | 2 (1·85) | 2 (1·96) | 1·0 |
| Constipation | 2 (1·85) | 1 (0·98) | 1·0 |
| Tremor | 1 (0·93) | 2 (1·96) | 0·96 |
| Diarrhea | 2 (1·85) | 0 (0·00) | 0·50 |
| Insomnia | 1 (0·93) | 1 (0·98) | 1·0 |
| Chest tightness | 1 (0·93) | 0 (0·00) | 1·0 |

**13. Supplementary Table S11. All adverse events that occurred during the trial period.**

| **Adverse events reported by patients** | | | |
| --- | --- | --- | --- |
| **Adverse events** | **Perospirone group**  **(N=108)** | **Placebo group**  **(N=102)** | **P value** |
|  | **No· (%) of patients** | |  |
| Somnolence | 8（7·41） | 10（9·80） | 0·54 |
| Dizziness | 11（10·19） | 4（3·92） | 0·078 |
| Nausea | 4（3·70） | 4（3·92） | 1·0 |
| Tiredness | 2（1·85） | 5（4·90） | 0·40 |
| Headache | 4（3·70） | 2（1·96） | 0·73 |
| Palpitation | 4（3·70） | 0（0·00） | 0·15 |
| Constipation | 3（2·78） | 2（1·96） | 1·0 |
| Tremor | 1（0·93） | 4（3·92） | 0·33 |
| Diarrhea | 2（1·85） | 2（1·96） | 1·0 |
| Insomnia | 3（2·78） | 2（1·96） | 1·0 |
| Chest tightness | 5（4·63） | 0（0·00） | 0·081 |
| **Adverse events rated by TESS** | | | |
| **Adverse events** | **Perospirone group**  **(N=99)** | **Placebo group**  **(N=96)** | **P value** |
|  | **No· (%) of patients** | |  |
| Toxic Delirium | 2 (2·02) | 1 (1·04) | 1·0 |
| Excitement/Agitation | 22 (22·22) | 17 (17·71) | 0·43 |
| Depressed Mood | 31 (31·31) | 33 (34·38) | 0·65 |
| Increased Activity | 17 (17·17) | 11 (11·46) | 0·26 |
| Decreased Activity | 15 (15·15) | 22 (22·92) | 0·17 |
| Insomnia | 30 (30·30) | 36 (37·50) | 0·29 |
| Somnolence | 40 (40·40) | 40 (41·67) | 0·86 |
| Hematologic Abnormality | 18 (18·18) | 16 (16·67) | 0·78 |
| Hepatic Function Abnormality | 22 (22·22) | 13 (13·54) | 0·11 |
| Urinalysis Abnormality | 30 (30·30) | 29 (30·21) | 0·99 |
| Muscular rigidity | 5 (5·05) | 4 (4·17) | 1·0 |
| Tremor | 22 (22·22) | 23 (23·96) | 0·77 |
| Dystonia | 1 (1·01) | 1 (1·04) | 1·0 |
| Akathisia | 24 (24·24) | 18 (18·75) | 0·35 |
| Dry Mouth | 41 (41·41) | 45 (46·88) | 0·44 |
| Nasal Congestion | 18 (18·18) | 14 (14·58) | 0·50 |
| Blurred Vision | 22 (22·22) | 18 (18·75) | 0·55 |
| Constipation | 24 (24·24) | 29 (30·21) | 0·35 |
| Increased Salivation | 5 (5·05) | 8 (8·33) | 0·36 |
| Sweating | 19 (19·19) | 22 (22·92) | 0·52 |
| Nausea/Vomiting | 18 (18·18) | 19 (19·79) | 0·77 |
| Diarrhea | 18 (18·18) | 13 (13·54) | 0·38 |
| Hypotension | 6 (6·06) | 3 (3·12) | 0·53 |
| Dizziness/Fainting | 28 (28·28) | 26 (27·08) | 0·85 |
| Tachycardia | 12 (12·12) | 14 (14·58) | 0·61 |
| Hypertension | 1 (1·01) | 0 (0·00) | 1·0 |
| ECG Abnormality | 19 (19·19) | 18 (18·75) | 0·94 |
| Skin Symptoms | 10 (10·10) | 13 (13·54) | 0·46 |
| Weight Gain | 25 (25·25) | 21 (21·88) | 0·58 |
| Weight Loss | 19 (19·19) | 24 (25·00) | 0·33 |
| Anorexia/Decreased Appetite | 18 (18·18) | 26 (27·08) | 0·14 |
| Headache | 24 (24·24) | 29 (30·21) | 0·35 |
| Tardive Dyskinesia | 1 (1·01) | 0 (0·00) | 1·0 |
| Other | 10 (10·10) | 13 (13·54) | 0·46 |

**14. Supplementary Table S12. Demographic and clinical characteristics of participants in the 4-Week and 4–8-Week subgroups at baseline.**

|  | **4-week group (n=133)** | **4–8-week group (n=44)** | **P value** |
| --- | --- | --- | --- |
| **Demographic characteristics** |  |  |  |
| Age (years), Mean±SD | 29·37±11·19 | 29·57±9·85 | 0·50 |
| Sex, Female no·(%) | 89 (66·9) | 26 (59·1) | 0·35 |
| Education (years), Mean±SD | 13·88±3·14 | 14·70±2·29 | 0·078 |
| **Psychiatric history** |  |  |  |
| Total duration of illness (months)  Mean±SD  Medium (P25,P75) | 47·01±50·40  24·00 (12·00, 72·00) | 43·55±44·27  24·00 (11·25, 60·00) | 0·71  0·98 |
| **Baseline severity of disease** |  |  |  |
| MADRS score, Mean±SD | 28·88±5·43 | 29·20±5·20 | 0·73 |
| QIDS-SR16 score, Mean±SD | 15·42±4·71 | 14·02±4·91 | 0·094 |
| HAMA score, Mean±SD | 18·74±7·20 | 21·86±7·23 | **0·014** |
| Q-LES-Q-SF score, Mean±SD | 37·85±8·00 | 39·84±10·37 | 0·30 |

Abbreviation: HAMA, Hamilton Anxiety Scale; MADRS, Montgomery-Åsberg Depression Rating Scale; QIDS-SR16, 16-item Quick Inventory of Depressive Symptomatology (Self-Report); Q-LES-Q-SF, Quality of Life Enjoyment and Satisfaction Questionnaire - Short Form;

**15. Supplementary Table S13. Efficacy outcomes (response and remission rates) in the 4-Week subgroup.**

| **Outcomes** | **Perospirone group (n=65)** | **Placebo group (n=68)** | **Odds ratio (95% CI)** | **P value** |
| --- | --- | --- | --- | --- |
| **Primary efficacy outcomes** | | | | |
| Response at Week 8, no·(%) | 43 (66·2) | 40 (58·9) | 1·368 (0·676-2·770) | 0·38 |
| Remission at Week 8, no·(%) | 35 (53·9) | 30 (44·1) | 1·488 (0·747-2·967) | 0·26 |
| **Secondary efficacy outcomes** | | | | |
| Response at Week 4, no·(%) | 35 (53·9) | 23 (33·8) | 2·286 (1·134-4·608) | 0·021 |
| Remission at Week 4, no·(%) | 26 (40·0) | 13 (19·1) | 2·886 (1·305-6·382) | 0·0090 |

**16. Supplementary Table S14. Efficacy outcomes (response and remission rates) in the 4–8-Week subgroup.**

| **Outcomes** | **Perospirone group (n=22)** | **Placebo group (n=22)** | **Odds ratio (95% CI)** | | **P value** |
| --- | --- | --- | --- | --- | --- |
| **Primary efficacy outcomes** | | | | | |
| Response at Week 8, no·(%) | 16 (72·7) | 13 (59·1) | 1·901 (0·531-6·809) | 0·32 | |
| Remission at Week 8, no·(%) | 13 (59·1) | 5 (22·7) | 4·967 (1·293-19·085) | 0·020 | |
| **Secondary efficacy outcomes** | | | | | |
| Response at Week 4, no·(%) | 13 (59·0) | 9 (40·9) | 2·126 (0·635-7·124) | 0·22 | |
| Remission at Week 4, no·(%) | 9 (40·9) | 5 (22·7) | 2·344 (0·630-8·718) | 0·20 | |

**17. Supplementary Table S15. Comparison of treatment effects on response and remission between the 4-Week and 4–8-Week subgroups.**

|  | **Week 8** | | **Week 4** | |
| --- | --- | --- | --- | --- |
|  | **Response (OR)** | **Remission (OR)** | **Response (OR)** | **Remission (OR)** |
| **Subgroup analyses** | | | | |
| 4-week group | 1·368 | 1·488 | 2·286 | 2·886 |
| 4–8-week group | 1·901 | 4·967 | 2·126 | 2·344 |
| **Overall analyses** | | | | |
| At least 4 weeks | 1·406 | 1·944 | 2·243 | 2·701 |

Overall analyses were conducted as the primary analysis, including participants who showed no response (<50% reduction in the MADRS total score) to at least one antidepressant with adequate dose and duration (≥4 weeks). Treatment effect was expressed as odds ratio (OR).

Abbreviation: OR, odds ratio;

**18. Supplementary Table S16. Tests of treatment-by-subgroup interaction for the primary efficacy outcomes.**

| **Variable** | **Estimate** | **SE** | **Wald χ²** | **DF** | **P value** |
| --- | --- | --- | --- | --- | --- |
| **Response at Week 8** | | | | | |
| Treatment group | 0·424 | 0·653 | 0·421 | 1 | 0·52 |
| Baseline MADRS score | 0·003 | 0·029 | 0·011 | 1 | 0·92 |
| Subgroup | -0·021 | 0·508 | 0·156 | 1 | 0·69 |
| Treatment×Subgroup | -0·110 | 0·745 | 0·022 | 1 | 0·88 |
| Intercept | 0·466 | 0·978 | 0·227 | 1 | 0·63 |
| **Remission at Week 8** | | | | | |
| Treatment group | 1·579 | 0·675 | 5·479 | 1 | 0·019 |
| Baseline MADRS score | -0·059 | 0·030 | 3·951 | 1 | 0·047 |
| Subgroup | 0·963 | 0·569 | 2·863 | 1 | 0·091 |
| Treatment×Subgroup | -1·179 | 0·761 | 2·397 | 1 | 0·12 |
| Intercept | 0·507 | 1·000 | 0·257 | 1 | 0·61 |

Subgroup was defined by the duration of antidepressant treatment: exactly 4 weeks vs. 4–8 weeks. The interaction term (Treatment×Subgroup) tests whether the treatment effect of adjunctive perospirone differs between subgroups. A statistically significant result (p < 0.05) indicates that the magnitude of benefit differs across subgroups, suggesting that the timing of adjunctive perospirone may influence treatment outcomes.

Abbreviation: DF, degress of freedom; SE, standard error; Wald χ²: Wald test statistic;

**19. Supplementary Table S17. Secondary efficacy outcomes (changes in rating scale scores) in the 4-Week subgroup.**

|  | **Week 4** | | |  | **Week 8** | | |  |
| --- | --- | --- | --- | --- | --- | --- | --- | --- |
|  | **Perospirone**  **group (n=65)** | **Placebo**  **group (n=68)** | **LSMD, vs.**  **placebo group**  **(95%CI)** | **P value** | **Perospirone**  **group (n=65)** | **Placebo**  **group (n=68)** | **LSMD, vs.**  **placebo group**  **(95%CI)** | **P value** |
|  | **LS mean (SE)** | **LS mean (SE)** |  |  | **LS mean (SE)** | **LS mean (SE)** |  |  |
| **Key secondary efficacy end points** | | | | | | | | |
| MADRS score | -14·19 (1·03) | -10·49 (1·01) | -3·70 (-6·24, -1·16) | 0·0046 | -16·54 (1·06) | -16·37 (1·03) | -0·17 (-2·78, 2·44) | 0·90 |
| QIDS-SR16 score | -6·98 (0·63) | -4·31 (0·61) | -2·67 (-4·18, -1·16) | 0·00070 | -6·89(0·70) | -6·25 (0·68) | -0·64 (-2·37, 1·09) | 0·47 |
| **Other secondary efficacy end points** | | | | | | | | |
| HAMA score | -7·73 (0·87) | -6·42 (0·85) | -1·32 (-3·46, 0·83) | 0·23 | -9·38 (0·81) | -8·98 (0·79) | -0·40 (-2·35, 1·54) | 0·68 |
| Q-LES-Q-SF score | 5·16 (1·07) | 3·29 (1·06) | 1·87 (-0·66, 4·39) | 0·15 | 7·35 (1·23) | 5·89 (1·21) | 1·46 (-1·56, 4·48) | 0·34 |

Abbreviation: HAMA, Hamilton Anxiety Scale; LS, least squares; LSMD, least squares mean difference; MADRS, Montgomery-Åsberg Depression Rating Scale; QIDS-SR16, 16-item Quick Inventory of Depressive Symptomatology (Self-Report); Q-LES-Q-SF, Quality of Life Enjoyment and Satisfaction Questionnaire - Short Form; SE, standard error.

**20. Supplementary Table S18. Secondary efficacy outcomes (changes in rating scale scores) in the 4–8-Week subgroup.**

|  | **Week 4** | | |  | **Week 8** | | |  |
| --- | --- | --- | --- | --- | --- | --- | --- | --- |
|  | **Perospirone**  **group (n=22)** | **Placebo**  **group (n=22)** | **LSMD, vs.**  **placebo group**  **(95%CI)** | **P value** | **Perospirone**  **group (n=22)** | **Placebo**  **group (n=22)** | **LSMD, vs.**  **placebo group**  **(95%CI)** | **P value** |
|  | **LS mean (SE)** | **LS mean (SE)** |  |  | **LS mean (SE)** | **LS mean (SE)** |  |  |
| **Key secondary efficacy end points** | | | | | | | | |
| MADRS score | -14·35 (1·71) | -9·34 (1·86) | -5·01 (-9·85, -0·17) | 0·043 | -18·13 (1·81) | -12·45 (1·96) | -5·67 (-10·82, -0·53) | 0·032 |
| QIDS-SR16 score | -5·73 (0·96) | -3·04 (1·11) | -2·69 (-5·37, -0·02) | 0·049 | -5·14(1·09) | -3·75 (1·24) | -1·38 (-4·45, 1·69) | 0·37 |
| **Other secondary efficacy end points** | | | | | | | | |
| HAMA score | -10·64 (1·39) | -7·42 (1·55) | -3·22 (-7·13, 0·69) | 0·10 | -12·86 (1·45) | -8·50 (1·62) | -4·37 (-8·47, -0·27) | 0·037 |
| Q-LES-Q-SF score | 3·63 (1·71) | -0·10 (1·97) | 4·63 (-0·11, 9·36) | 0·056 | 3·27 (1·96) | 0·58 (2·22) | 2·68 (-2·82, 8·19) | 0·33 |

Abbreviation: HAMA, Hamilton Anxiety Scale; LS, least squares; LSMD, least squares mean difference; MADRS, Montgomery-Åsberg Depression Rating Scale; QIDS-SR16, 16-item Quick Inventory of Depressive Symptomatology (Self-Report); Q-LES-Q-SF, Quality of Life Enjoyment and Satisfaction Questionnaire - Short Form; SE, standard error.

**21. Supplementary Table S19. Comparison of treatment effects on secondary efficacy outcomes (changes in rating scale scores) between the 4-Week and 4–8-Week subgroups.**

|  | **Week 8** | | | | **Week 4** | | | |
| --- | --- | --- | --- | --- | --- | --- | --- | --- |
|  | **MADRS (LSMD)** | **QIDS-SR16 (LSMD)** | **HAMA (LSMD)** | **Q-LES-Q-SF (LSMD)** | **MADRS (LSMD)** | **QIDS-SR16 (LSMD)** | **HAMA (LSMD)** | **Q-LES-Q-SF (LSMD)** |
| **Subgroup analyses** | | | | | | | | |
| 4-week group | -0·17 | -0·64 | -0·40 | 1·46 | -3·70 | -2·67 | -1·32 | 1·87 |
| 4–8 week group | -5·67 | -1·38 | -4·37 | 2·68 | -5·01 | -2·69 | -3·22 | 4·63 |
| **Overall analyses** | | | | | | | | |
| At least 4 weeks | -1·83 | -0·85 | -1·86 | 2·17 | -4·37 | -2·65 | -2·16 | 2·64 |

Overall analyses were conducted as the primary analysis, including participants who showed no response (<50% reduction in the MADRS total score) to at least one antidepressant with adequate dose and duration (≥4 weeks). Treatment effect was expressed as least squares mean difference (LSMD).

Abbrevation: HAMA, Hamilton Anxiety Scale; LSMD, least squares mean difference; MADRS, Montgomery-Åsberg Depression Rating Scale; QIDS-SR16, 16-item Quick Inventory of Depressive Symptomatology (Self-Report); Q-LES-Q-SF, Quality of Life Enjoyment and Satisfaction Questionnaire - Short Form;

**22. Data Sharing Statement**

Efficacy and safety of perospirone as adjunctive therapy in major depressive disorder patients with inadequate response to antidepressants: a randomized clinical trial

**Data**

**Additional Information:** Chinese Clinical Trial Registry Identifier: ChiCTR2200063354

**Data available:** Yes

**Data types:** Deidentified participant data

**How to access data:** Contact the corresponding authors: Yan Zhang, yan.zhang@csu.edu.cn; Lingjiang Li, LLJ2920@csu.edu.cn.

**When available:** With publication

**Supporting Documents**

**Document types:** None

**Additional Information**

**Who can access the data:** Researchers whose proposed use of the data has been approved.

**Types of analyses:** Specified purpose

**Mechanisms of data availability:** With investigator support, after approval of a proposal, with a signed data access agreement.
